# Biochemical and structural features of diverse bacterial glucuronoyl esterases facilitating recalcitrant biomass conversion

**DOI:** 10.1186/s13068-018-1213-x

**Published:** 2018-08-01

**Authors:** Jenny Arnling Bååth, Scott Mazurkewich, Rasmus Meland Knudsen, Jens-Christian Navarro Poulsen, Lisbeth Olsson, Leila Lo Leggio, Johan Larsbrink

**Affiliations:** 1 Wallenberg Wood Science Center, Division of Industrial Biotechnology, Department of Biology and Biological Engineering, Chalmers University of Technology, Gothenburg, Sweden; 2 Department of Chemistry, University of Copenhagen, Copenhagen, Denmark

**Keywords:** Glucuronoyl esterase, Carbohydrate esterase, CE15, Carbohydrate-active enzyme, Biomass conversion, Lignin–carbohydrate complexes, Xylan

## Abstract

**Background:**

Lignocellulose is highly recalcitrant to enzymatic deconstruction, where the recalcitrance primarily results from chemical linkages between lignin and carbohydrates. Glucuronoyl esterases (GEs) from carbohydrate esterase family 15 (CE15) have been suggested to play key roles in reducing lignocellulose recalcitrance by cleaving covalent ester bonds found between lignin and glucuronoxylan. However, only a limited number of GEs have been biochemically characterized and structurally determined to date, limiting our understanding of these enzymes and their potential exploration.

**Results:**

Ten CE15 enzymes from three bacterial species, sharing as little as 20% sequence identity, were characterized on a range of model substrates; two protein structures were solved, and insights into their regulation and biological roles were gained through gene expression analysis and enzymatic assays on complex biomass. Several enzymes with higher catalytic efficiencies on a wider range of model substrates than previously characterized fungal GEs were identified. Similarities and differences regarding substrate specificity between the investigated GEs were observed and putatively linked to their positioning in the CE15 phylogenetic tree. The bacterial GEs were able to utilize substrates lacking 4-OH methyl substitutions, known to be important for fungal enzymes. In addition, certain bacterial GEs were able to efficiently cleave esters of galacturonate, a functionality not previously described within the family. The two solved structures revealed similar overall folds to known structures, but also indicated active site regions allowing for more promiscuous substrate specificities. The gene expression analysis demonstrated that bacterial GE-encoding genes were differentially expressed as response to different carbon sources. Further, improved enzymatic saccharification of milled corn cob by a commercial lignocellulolytic enzyme cocktail when supplemented with GEs showcased their synergistic potential with other enzyme types on native biomass.

**Conclusions:**

Bacterial GEs exhibit much larger diversity than fungal counterparts. In this study, we significantly expanded the existing knowledge on CE15 with the in-depth characterization of ten bacterial GEs broadly spanning the phylogenetic tree, and also presented two novel enzyme structures. Variations in transcriptional responses of CE15-encoding genes under different growth conditions suggest nonredundant functions for enzymes found in species with multiple CE15 genes and further illuminate the importance of GEs in native lignin–carbohydrate disassembly.

**Electronic supplementary material:**

The online version of this article (10.1186/s13068-018-1213-x) contains supplementary material, which is available to authorized users.

## Background

**Fig. 1:**
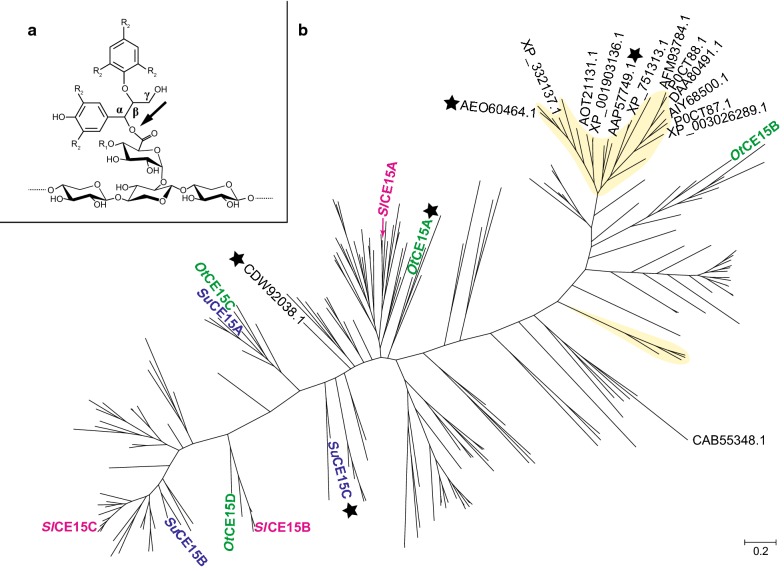
**a** General structure of LCC esters (either α- or γ-linked to glucuronic acid moieties on xylan), and site of enzymatic cleavage by glucuronoyl esterases (arrow). R_1_ may be either H or a methyl moiety, while R_2_ labels represent possible further connections to the lignin network. **b** Phylogenetic tree of all CE15 catalytic domains in CAZy. Biochemically characterized members are labeled with their respective Genbank accession numbers. Branches representing members of fungal origin are shaded in yellow. Stars indicate structurally determined members. Enzymes characterized in this study are labeled with their protein names, color coded in green for *O. terrae*, magenta for *S. linguale*, and blue for *S. usitatus*

Deconstruction of the plant cell wall represents a significant challenge for microorganisms as the network of interlinked cellulose fibers, hemicelluloses, and lignin is highly recalcitrant to enzymatic attack. A feature of the plant cell wall that adds to its recalcitrance, but rather poorly characterized and understood, is the presence of covalent bonds between polysaccharides and lignin, the so-called lignin–carbohydrate complexes (LCCs) [1]. Three types of covalent LCC bonds have been identified: ester, ether, and glycosidic. Of these, enzymatic cleavage has to date only been proposed for the LC ester bonds, found between 4-*O*-methyl-glucuronoyl moieties of xylan and the alcohol moieties of lignin (Fig. 1a). The enzymes proposed to cleave these are glucuronoyl esterases (GEs), found in carbohydrate esterase family 15 (CE15) in the carbohydrate-active enzymes database (CAZy; http://www.cazy.org; [2]). Since the characterization of the first GE from *Schizophyllum commune* [3, 4], CE15 members from a multitude of biomass-degrading microorganisms have been identified, exhibiting as low as 15% sequence identity [5]. Despite the proposed importance of CE15 enzymes in LCC cleavage, only a handful of GEs have been biochemically characterized, and only three enzymes have been structurally characterized.

To date, 11 fungal [3, 6–15] and 2 bacterial GEs have been biochemically characterized [16, 17] using alkyl and alkyl aryl alcohol esters of 4-*O*-methyl glucuronic acid of varying complexity. An equatorial configuration of the C4 hydroxyl moiety has been proposed to be important for GE activity as fungal GEs have been reported to exclusively attack esters of d-glucuronic acid (GlcA) and not d-galacturonic acid (GalA) [6, 8]. Furthermore, studies of fungal GEs indicate that methylation of the C4 hydroxyl moiety is crucial for enzymatic activity. The two bacterial GEs described to date exhibit broader substrate specificities than studied fungal enzymes, with acetyl esterase activity detected on a variety of substrates [17, 18]. Regarding activity on native lignocellulosic material, the recent investigation of *Aa*GE1 from the fungus *Acremonium alcalophilum* was the first report showing direct enzymatic LC ester cleavage of wood-extracted LCC fractions [19]. The role(s) of GEs on lignocellulose degradation has further been implied by somewhat improved saccharification of corn fiber by addition of fungal GEs to commercial hydrolytic enzyme cocktails [20], and recently the fungal *Cerrena unicolor Cu*GE was shown to release aldouronic acid products and act synergistically with a GH10 *endo*-xylanase on birchwood lignin precipitates [21].

Two fungal and one bacterial CE15 protein structures have been solved: Cip2 from *Hypocrea jecorina* (PDB: 3pic), *St*GE2 from *Myceliophtora thermophila* (PDB: 4g4g), and MZ0003, a bacterial CE15 cloned from a marine metagenomic library (PDB: 6ehn) [22–24]. The enzymes are α/β-hydrolases with a catalytic triad typical of esterases. MZ0003 is distinct from the fungal structures, by having a deeper substrate binding pocket, and the residue providing the acidic functionality of the catalytic triad is found on a different loop. The only CE15 structure with a ligand is an active site mutant of *St*GE2, co-crystallized with the methyl ester of 4-*O*-methyl glucuronoate (PDB: 4g4j) [23].

Interestingly, many microorganisms encode several CE15 proteins, indicating potential differences in both substrate specificities and physiological functions. To gain insights into the biological roles of CE15 enzymes that are diverse in primary sequence, we performed detailed analyses of ten unique CE15 members from three bacterial species. The targets, exhibiting sequence identities as low as 25%, were biochemically characterized using a range of model substrates, and the three-dimensional structures were solved for two of the enzymes. We investigated the regulation of CE15 gene expression in response to different carbon sources for one of the species, and furthermore demonstrated the potential of GEs to enhance hydrolysis of native (non-pretreated) lignocellulosic material, by supplementing a commercial hydrolytic enzyme cocktail with GEs, which resulted in significantly improved saccharification. These findings provide novel insights into the diversity, substrate specificities, structural differences, and activity on biomass-derived substrates of bacterial members across the CE15 family.

## Results

### Phylogenetic analysis

Since its creation over a decade ago, the CE15 family remains largely unexplored, with most studies having focused on fungal enzymes, despite a much greater number of bacterial members. To investigate the sequence diversity of the family, a phylogenetic tree of all CE15 catalytic domains was constructed (Fig. 1b; Additional file 1: Figure S1). CE15 is currently a small family (239 entries in CAZy, July 2018) and the phylogeny illustrates a high sequence divergence with many deeply rooted lineages. The tree revealed apparent separations of fungal and bacterial members, with fungal enzymes clustering into a major and a minor clade. All hitherto-characterized fungal enzymes fall into the major clade, which is also most dissimilar to the majority of bacterial members regarding primary protein structure. Being a small family with members exhibiting high sequence diversity (as low as 25% identity), branching in sections of the tree could, however, not be strongly supported by bootstrap analysis (Additional file 1: Figure S1). The two previously partially characterized bacterial enzymes were found in the middle section of the tree, which leaves the majority of the tree completely unexplored. To explore the diversity of CE15, and possibly unravel new functionalities, ten bacterial enzymes spanning the phylogenetic tree were selected for in-depth studies. The targets were selected from three bacterial species from different habitats where active decomposition of plant biomass occurs, and which all encode multiple putative CE15 enzymes: *Opitutus terrae* (anaerobic, isolated from anoxic rice paddy soil, Italy; 4 enzymes [25]); *Spirosoma linguale* (aerobic, isolated from lab water bath, found globally in freshwater and soil environments; 3 enzymes [26]), and *Solibacter usitatus* (aerobic, pasture soil, Australia; 3 enzymes [27]). The genomes of the selected organisms do not encode additional CE15 enzymes, and further, all targets were distinctly different in primary sequence (Additional file 1: Table S1). Why certain species encode multiple distinct CE15 enzymes (to date, ≤ 4 CE15 genes in a single genome [2]) is unclear, and the choice of targets enabled analysis of potential different biological roles.

### Biochemical characterization and substrate specificity

The activities of the ten *O. terrae*, *S. linguale*, and *S. usitatus* CE15 enzymes were assayed on a range of substrates, and kinetic parameters determined where possible (Table 1; Additional file 1: Table S2, Figure S2). All enzymes rapidly cleaved the ester bond in benzyl glucuronoate (BnzGlcA), which was used to determine their respective pH dependencies. The *S. linguale* enzymes differed from the *O. terrae* and *S. usitatus* enzymes by exhibiting lower pH optima (pH 5.5–6.5 vs. 7–8.5) (Additional file 1: Figure S3). Three of the enzymes, *Ot*CE15D, *Su*CE15A & C, exhibited exceptionally high catalytic efficiencies (in the 10^4^ s^−1^ M^−1^ range) on BnzGlcA (Table 1), which is 10 to 100-fold higher than reported for fungal GEs [9, 13, 15, 28]. *Su*CE15C also exhibited high catalytic efficiency on allyl- and methyl-substituted glucuronoate esters (AllylGlcA and MeGlcA, respectively). No kinetic parameters have previously been reported for bacterial GEs which limits direct comparisons.

**Table 1:** Catalytic efficiencies of the investigated CE15 enzymes on model substrates

Enzyme/substrate	*k*_cat_/*K*_m_ (s^−1^M^−1^)^a^				
	BnzGlcA	AllylGlcA	MeGlcA	MeGalA	*p*Np-Ac
*Ot*CE15A	4.64 × 10^3^	8.80 × 10^3^	6.85 × 10^3^	4.85 × 10^3^	3.23 × 10^1^
*Ot*CE15B	1.86 × 10^1^	2.82	1.14	8.68	2.56
*Ot*CE15C	1.16 × 10^4^	2.49 × 10^3^	8.98 × 10^2^	1.19 × 10^3^	3.97 × 10^1^
*Ot*CE15D	1.11 × 10^4^	3.45 × 10^3^	5.19 × 10^2^	1.95 × 10^−6^	9.51
*Sl*CE15A	1.88 × 10^3^	1.00 × 10^3^	1.55 × 10^3^	3.82 × 10^1^	3.09 × 10^1^
*Sl*CE15B	2.60 × 10^3^	9.08 × 10^2^	4.57 × 10^2^	3.66 × 10^−7^	ND^b^
*Sl*CE15C	9.69 × 10^1^	1.11 × 10^2^	1.03 × 10^2^	3.73 × 10^−6^	ND^b^
*Su*CE15A	2.20 × 10^4^	5.47 × 10^3^	2.32 × 10^3^	1.62 × 10^3^	5.09
*Su*CE15B	1.49 × 10^3^	3.65 × 10^2^	6.00 × 10^−2^	9.00 × 10^−3^	1.82 × 10^1^
*Su*CE15C	2.27 × 10^4^	1.57 × 10^4^	1.66 × 10^4^	1.59 × 10^3^	1.09 × 10^1^

Benzyl (Bnz), allyl (Allyl) and methyl (Me) esters of glucuronoate (GlcA) and galacturonoate (GalA), and 4-nitrophenol acetate (*p*NP-Ac)

^a^SEM of duplicate measurements are presented in Additional files 1

^b^Not determined due to activities below the detection limit

*K*_m_ values for fungal GEs acting on BnzGlcA have consistently been reported in the millimolar range, albeit with large variations (~2–80 mM). The bacterial CE15 enzymes investigated here displayed *K*_m_ values at a much lower and narrow range, with four enzymes (*Ot*CE15C & D, *Sl*CE15B, and *Su*CE15A) reaching sub-millimolar values (0.4–0.6 mM; Additional file 1: Table S2). Sub-millimolar *K*_m_ values have for fungal CE15 members only been observed for few cases on 4-*O*-methylated esters of glucuronic acid [6, 8, 29]; substrates with this substitution are not commercially available. The remarkably low *K*_m_ values of the bacterial enzymes indicate that the 4-*O*-methyl substitution on BnzGlcA is not a strict requirement for all CE15 enzymes which possibly reflects potential variability in biomass substrate structure.

Most of the enzyme targets exhibited minimal discrimination between the ester substituents on glucuronate moieties (benzyl, allyl, or methyl; Additional file 1: Figure S2, Table S2). However, several enzymes (Fig. 1b), i.e., *Ot*CE15C & D, *Sl*CE15B, and *Su*CE15A, displayed tenfold increases in *K*_m_ for AllylGlcA and MeGlcA compared to BnzGlcA. *Ot*CE15A & C, and *Su*CE15A, further exhibited little to no discrimination between methyl esters of glucuronoate versus galacturonoate (MeGalA; Table 1; Additional file 1: Table S2), contrasting especially *Ot*CE15D and *Sl*CE15B & C, which exhibited 10^7–9^-fold lower *k*_cat_/*K*_m_ values for MeGalA. Enzymes with comparable or higher activity on MeGalA versus MeGlcA were assayed for pectin methyl esterase activity, but no activity (MeOH release) could be detected.

Acetyl esterase activity, mainly on 4-nitrophenyl acetate (*p*NP-Ac), has been reported with MZ0003 [17]. The CE15 enzymes investigated here displayed only trace activity on *p*NP-Ac, with ~ 1000-fold lower *k*_cat_/*K*_m_ values compared to MZ0003 (Table 1). Acetyl xylan esterase activity was investigated on 1,2,3,4-tetra-*O*-acetyl-β-d-xylopyranose (TetAcXyl) and biomass (ball milled corn cob and Japanese beech) using the four *O. terrae* enzymes which apparently span the phylogenetic tree. The *k*_cat_/*K*_m_ values of the enzymes were as low or lower on TetAcXyl as on *p*NP-Ac (Additional file 1: Table S2), and no liberated acetate was detected after prolonged incubation of the enzymes with biomass, indicating that the enzymes are GEs without significant acetyl esterase activity.

### Structural determinations

#### Overall structure

As structural information regarding CE15 enzymes is sparse, with only two fungal and one bacterial structure solved to date [22–24], structural determination of all the CE15 members investigated here was pursued using X-ray crystallography. Structures for two CE15 enzymes were solved by SAD phasing (seleno-l-methionine for *Su*CE15C, PDB ID 6gu8; and gold-derivatized *Ot*CE15A, PDB ID 6grw) and subsequent molecular replacement (native *Su*CE15C and *Ot*CE15A,; PDB ID 6gry and 6gs0, respectively).

**Fig. 2:**
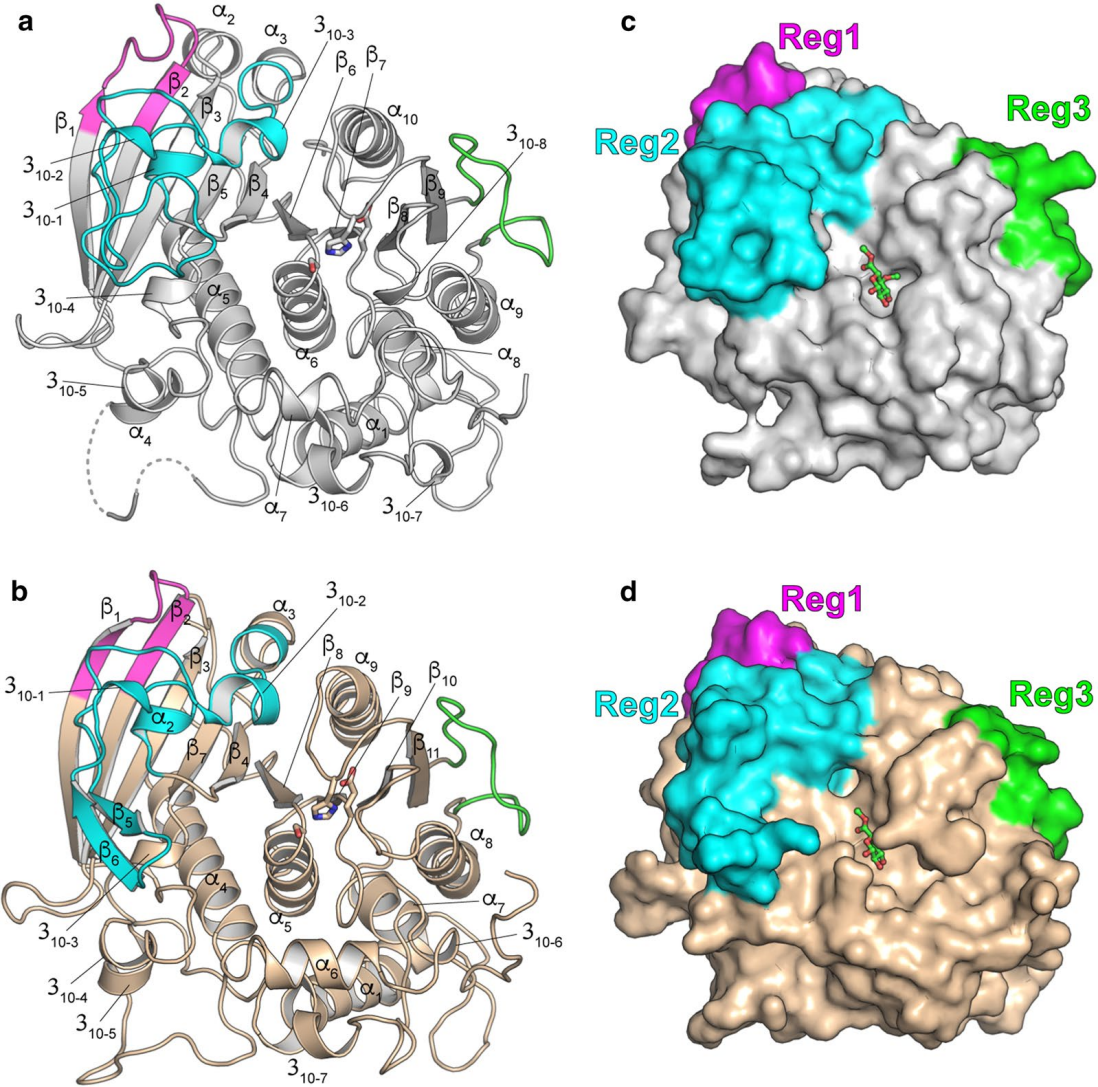
Overall structures of *Ot*CE15A (**a**) and *Su*CE15C (**b**). The catalytic triad (Ser-His-Glu) for each enzyme is shown as sticks. The inserted regions 1, 2, and 3 relative to the fungal CE15 enzymes are colored magenta, cyan, and green, respectively. Space filling representations of *Ot*CE15A (**c**) and *Su*CE15C (**d**), highlighting the inserted regions relative to the methyl ester of 4-*O*-methyl glucuronoate substrate, generated from structural alignment with the co-crystallized structure of *St*GE2 (PDB: 4G4J)

The overall folds of *Ot*CE15A and *Su*CE15C conform to the α/β-hydrolase fold of the previously solved CE15 structures [Cα root mean square deviation of 2.2 Å to 3pic and 4g4g over 304 residues; and 1.4 Å to 6ehn over 372 residues for *Ot*CE15A; 2.6 Å to 3pic; 2.7 Å to 4g4g over 305 residues; and 2.3 Å to 6ehn over 377 residues for *Su*CE15C (30)], consisting of a three-layer αβα sandwich with a solvent-exposed cleft comprising the active site and catalytic residues (Fig. 2). A single molecule was found in the asymmetric units of both *Ot*CE15A and *Su*CE15C, and crystal contact analysis with PISA [30] supported a monomeric state in solution consistent with gel filtration analysis (data not shown).

#### Comparison of overall structure

Similar to the recently solved structure of MZ0003, both *Ot*CE15A and *Su*CE15C differ from the fungal structures by the presence of 3 inserted regions (Reg1–3; Fig. 2c, d). Reg1 comprises 12 residues which extend β1, β2, and the loop between the strands. The sequence identity between the MZ0003, *Ot*CE15A, and *Su*CE15C in this region is high (≥ 80%) and the loop region packs against a portion of Reg2. Like MZ0003, Reg2 in *Ot*CE15A and *Su*CE15C comprises 40–45 residues originating from β4 and contains a few short helical segments and, in the case of *Su*CE15C, two β strands which form a slightly misaligned antiparallel sheet. Reg2 packs against the face of the main β-sheet, extends across an α-helix (α5 in *Ot*CE15A and α4 in *Su*CE15C), and packs against Reg1 forming a contiguous unit between the two regions. Reg3 is a 15-residue hydrophilic loop found between, and packing against, the last β-strand of the main sheet and an α-helix (α_9_ and α_8_ in *Ot*CE15A and *Su*CE15C, respectively) (Fig. 2a, b).

#### Comparison of active site pockets

**Fig. 3:**
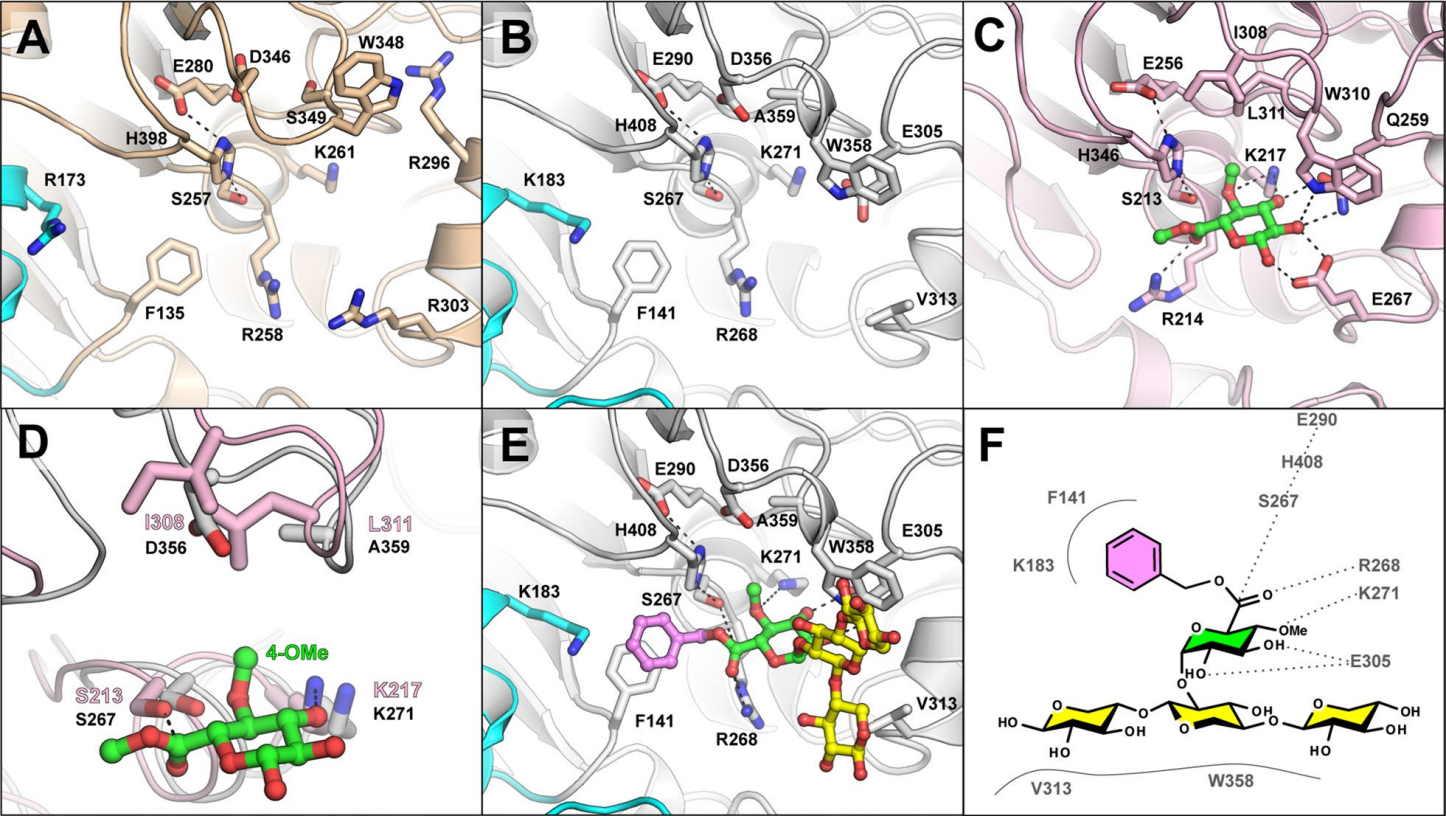
Active site organization and docking simulation. Comparison of active site pockets of the *Su*CE15C (**A**), *Ot*CE15A (**B**), and *St*GE2 (**C**). The methyl ester of 4-*O*-methyl glucuronoate co-crystallized with *St*GE2 is shown in green sticks. The region around the binding site of the 4-*O*-methyl substituent in *St*GE2 (pink) and *Ot*CE15A (grey) highlights a common aspartate in bacterial structures that contrasts the hydrophobic pocket of fungal enzymes (**D**). Representative docking simulation of *Ot*CE15A with a benzyl ester of 4-*O*-methyl-glucuronoxylotriose (**E**) with proposed interactions indicated (**F**)

The catalytic triad (Ser/His/Glu) and most of the residues shown to interact with the glucuronoate ester moiety in the previously solved fungal *St*GE2 structure [23] are conserved in *Ot*CE15A, *Su*CE15C, and the previously determined MZ0003 (Fig. 3A–C; Additional file 1: Figure S4). The tryptophan residue in *St*GE2 (Trp310) found hydrogen bonding to the 2-OH of the ligand is conserved in the bacterial structures (Trp358 in *Ot*CE15A, Trp348 in *Su*CE15C, and Trp334 in MZ003), whereas Glu267 in *St*GE2 which hydrogen bonds with both 2-OH and the anomeric hydroxyl group (in β configuration) is not conserved (Arg303 in *Su*CE15C, Val313 in *Ot*CE15A, and Ser289 in MZ0003). Gln259 of *St*GE2 is observed hydrogen bonding with both the 2-OH and 3-OH moieties of the sugar ring, and the equivalently positioned residues (with respect to main chain) in *Ot*CE15A, *Su*CE15C, and MZ0003 are Glu305, Glu295, and Glu281, respectively. However, in *Su*CE15C, the loop containing Glu295 is modeled with the acidic side chain rotated away from the active site, and instead Arg296 occupies the equivalent space.

An arginine in *St*GE2 (Arg214), found beside the catalytic serine (Ser213), is proposed to form the stabilizing oxyanion hole during catalysis through the interaction of the main chain amino group and the carbonyl of the co-crystallized 4-*O*-methyl glucuronoate (3.4 Å distance) [23]. An arginine is found at the same position in *Ot*CE15A (Arg268), *Su*CE15C (Arg258), and MZ0003 (Arg244), and is conserved in all CE15 enzymes characterized to date (Additional file 1: Figure S5), except *Ot*CE15B. *Ot*CE15B has a tyrosine in the equivalent position, and the markedly reduced catalytic activity of the enzyme versus other GEs may be a result of the Arg → Tyr substitution (Table 1). Further analysis of *St*GE2 reveals that, in addition to the main chain amino group, the *N*^*η*1^ of the Arg214 guanidinium moiety is positioned close to the substrate carbonyl (3.5 Å distance), which together with the sequence conservation and crippled activity of *Ot*CE15B suggests an important role of the guanidinium functionality in catalytically competent CE15 members.

The lysine residue (Lys217) in *St*GE2, noted to interact with the 4-*O*-Me oxygen of the ligand is conserved in the bacterial structures (Lys271 in *Ot*CE15A, Lys261 in *Su*CE15C, and Lys247 in MZ0003). A small hydrophobic patch in *St*GE2 (Ile308 and Leu311) possibly aids in positioning the 4-*O*-Me moiety (Fig. 3D), and in all fungal enzymes characterized to date, the leucine is conserved while various hydrophobic residues are found at the position of the isoleucine (Additional file 1: Figure S5). In the bacterial structures, small residues are found in the equivalent leucine position (Ala359 in *Ot*CE15A, Ser349 in *Su*CE15C, and Ala335 in MZ0003) while an aspartate residue is found in the same position as the isoleucine (Asp356 in *Ot*CE15A, Asp346 in *Su*CE15C, and Asp332 in MZ0003) (Fig. 3D; Additional file 1: Figure S4). Of the bacterial enzymes characterized here, all but three contain a small residue and an aspartate in the aforementioned positions (Additional file 1: Figure S5). *Ot*CE15B, closest to the fungal clades (Fig. 1), and the *Su*CE15B and *Sl*CE15C enzymes, furthermost from the fungal members, maintain the fungal leucine and hydrophobic residue pattern. The presence of an aspartate in this position likely affects binding of substrates containing 4-*O*-Me moieties and may facilitate binding to unmethylated substrates, such as the model substrates utilized here.

A small cleft within the pocket formed by Reg2 contains a conserved phenylalanine residue (Phe141 in *Ot*CE15A, Phe135 in *Su*CE15C, and Phe117 in MZ0003) and a basic residue (Lys183 in *Ot*CE15A, Arg173 in *Su*CE15C, and Arg160 in MZ0003) (Fig. 3A, B; Additional file 1: Figure S4). The phenylalanine side chain is located behind the catalytic serine and between the catalytic histidine and the conserved arginine. In *St*GE2, the methyl group of the co-crystallized ester projects toward this position, suggesting possible binding interactions with aromatic lignin substituents. Of the bacterial enzymes characterized here, all but two contain a phenylalanine at the same position, by primary sequence alignment, while the two others contain a phenylalanine close in primary sequence which may fulfill a similar functionality (Additional file 1: Figure S5).

#### Docking simulations

To investigate putative lignin- and xylan-binding sites in our solved structures, docking simulations of *Ot*CE15A and *Su*CE15C with a benzyl ester of 4-*O*-methyl-glucuronoxylotriose (glucuronoate α-1,2 linked to the middle xylose residue) were performed. Several binding poses presumed compatible with catalytic activity were observed. Consistent among these was the placement of the benzyl moiety toward, and sometimes stacking on top of, the conserved bacterial phenylalanine of Reg2 (Fig. 3F). The cleft formed by Reg2 is larger than a benzyl moiety, and lined with hydrophilic residues, which could possibly accommodate and provide specificity for larger lignin fragments containing multiple hydroxyl groups. The positioning of the xylotriose portion of the ligand was more variable but consistently spanned an α-helix (α_7_ in *Ot*CE15A and α_6_ in *Su*CE15C) and stacked against a tryptophan residue (Trp358 in *Ot*CE15A and Trp348 in *Su*CE15C). This tryptophan is conserved among all the characterized CE15 members, and in both *St*GE2 and the docked structures is found hydrogen bonding the glucuronoate 2′-OH with its Nε atom (Additional file 1: Figure S5). The docking simulations suggest that CE15 members can accommodate and possibly bind xylose residues proximal to the glucuronic acid of either glucuronoxylan or smaller oligosaccharides.

### Transcriptional analysis of *S. linguale* CE15-encoding genes

**Fig. 4:**
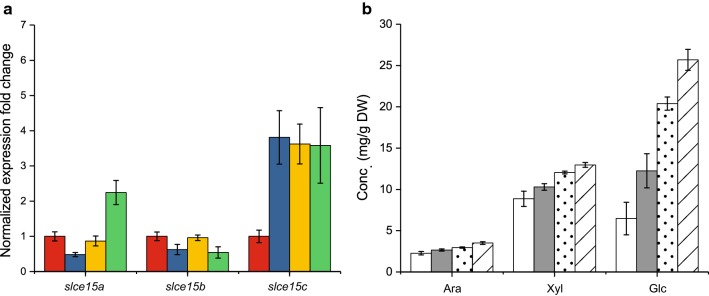
**a** Change in CE15 gene expression (*slce15A*, *slce15B,* and *slce15C*) in *S. linguale* cells grown on xylose (blue), corn cob xylan (yellow), and corn cob biomass (green) normalized to growth on glucose (red). The RNA polymerase sigma factor *rpoD* (locus tag Slin_1987) served as reference gene. Error bars indicate the SEM of triplicate measurements. **b** Release of monosaccharides (arabinose, xylose, and glucose) after 24-h enzymatic hydrolysis of ball-milled corn cob using Ultraflo^®^, without the addition of CE15 (white), or with the addition of *Sl*CE15A (gray), *Su*CE15A (dotted), or *Su*CE15C (striped). Error bars represent the SEM of triplicate measurements

Several microorganisms encode multiple CE15 members, but the biological reason for this is unknown. To assess whether transcriptional differences exist in bacteria that encode multiple CE15 genes, *S. linguale* (the single cultivable species of the three investigated) was grown on glucose, xylose, corn cob xylan, and milled corn cob biomass, and its transcription of CE15 genes was monitored by quantitative reverse transcription PCR (RT-qPCR) relative to the RNA polymerase sigma factor *rpoD* (Fig. 4a). While *slce15b* was constitutively expressed in all growth conditions, expression of *slce15a* was similar for glucose, xylose, and xylan, but increased twofold on milled corn cob, and *slce15c* showed a three to fourfold increase in response to any xylose-containing carbon source compared to glucose. The expression of the *Sl*CE15A-, B-, and C-encoding genes are thus apparently regulated by different biological cues. Together with the biochemical data, this differential regulation indicates nonredundancy and different roles of the CE15 enzymes in the biology of *S. linguale*.

### CE15 enzymes enhance the hydrolysis of corn cob

Cleavage of the ester linkages found in LCCs could aid enzymatic saccharification of biomass through selective de-coupling of lignin from polysaccharides. To investigate possible boosting of lignocellulose hydrolysis through bacterial GE action, selected CE15 enzymes were added to the cellulo- and hemicellulolytic cocktail Ultraflo^®^ during hydrolysis of ball milled corn cob (no further pretreatment was performed to limit disruption of the chemical structure). Corn cobs are abundant industrial waste streams, consisting chiefly of cellulose (47% dry weight; dw), but are also rich in complex heteropolysaccharides (heteroxylans, 28% dw, and arabinan, 5% dw) [31]. The GEs *Sl*CE15A, *Su*CE15A or *Su*CE15C were selected to supplement hydrolysis reactions based on their high activity on BnzGlcA at pH 5.5 (recommended for Ultraflo^®^) together with their high long-term stabilities. Increased concentrations of glucose, xylose, and arabinose were observed in all GE-supplemented reactions (Fig. 4b). Released arabinose and xylose increased moderately (20–50%), whereas the glucose concentration increased dramatically by 90–300%. *Su*CE15C was consistently the most efficient boosting enzyme, followed by *Su*CE15A and *Sl*CE15A, somewhat reflecting their activity levels on BnzGlcA (Table 1; Additional file 1: Table S2). The increases of released monosaccharides strongly suggest an important role of CE15 enzymes in facilitating more efficient substrate access for a range of classical polysaccharide-degrading enzymes.

## Discussion

Glucuronoyl esterases have been suggested to play a crucial role in separating carbohydrates from lignin in lignocellulose. Direct evidence of the biological role(s) of these enzymes is still speculative, due to a lack of suitable analytical methods on native biomass and extracted LCCs. The ten bacterial enzymes investigated here have significantly higher catalytic efficiencies and wider substrate ranges on model substrates than previously characterized CE15 enzymes, suggesting abilities to act on diverse natural substrates. The similarities and differences in substrate specificities among the CE15 enzymes appeared to correspond to the enzymes’ location in the phylogenetic tree, and the transcriptional analyses support the hypothesis of nonredundant roles of the gene copies within bacterial species encoding multiple CE15 enzymes.

Previous studies have demonstrated that fungal GEs have strict substrate specificities for glucuronoate esters and require methyl substitutions on the 4-OH of the glucuronoate for full activity [9, 15, 32]. Several bacterial enzymes seem to lack these constraints and display the highest recorded *k*_cat_/*K*_m_ values of GEs to date on the BnzGlcA model substrate, lacking 4-OH methylation. Further, the majority of the studied bacterial enzymes had similar *K*_m_ values and high catalytic efficiencies on all GlcA ester substrates, regardless of the alcohol portion. Several did not discriminate between MeGlcA and MeGalA demonstrating that CE15 members are not restricted to act on GlcA-derived esters as previously claimed. The hydrophobic patch observed in fungal structures, which may support 4-*O*-Me positioning, is changed in many bacterial enzymes to a more open and hydrophilic region that may contribute to more promiscuous substrate specificities. However, comparison of the sequences and kinetics of all characterized enzymes in the present study does not completely correlate with this observation. In particular, *Ot*CE15D and *Sl*CE15B, possessing equivalent residues as *Ot*CE15A and *Su*CE15C in this region, were unable to be saturated with MeGalA, indicating that other determinants contribute to uronate discrimination.

The structural determinations of *Ot*CE15A and *Su*CE15C reveal similar overall folds to previously determined CE15 structures, with closest structural similarity to the recently released bacterial structure of MZ0003 [22–24]. Of the three inserted regions found in the bacterial structures, Reg1 and Reg2 form a contiguous unit proximal to the active site, and sequence analysis suggests that, although with variation of length and sequence diversity, these inserted regions may be conserved among bacterial CE15 members. Catalytically competent docking poses consistently positioned the benzyl ring of glucuronoate esters near, or stacking with, a phenylalanine conserved among most bacterial CE15 members, suggesting a previously unidentified binding site for the (lignin-derived) alcohol moiety in LCC substrates. Several GEs, from both fungal and bacterial origins, have marked preferences for larger ester substituents, such as having improved kinetic parameters for BnzGlcA versus MeGlcA, which supports the hypothesis that molecular determinants to facilitate binding of larger lignin fragments exist within some GEs [9, 13]. Due to the lack of larger ligands in CE15 structures, identification of residues providing specificity, or tolerance, to lignin fragments remains tentative. However, the large ridges inserted proximal to the active site in bacterial structures constitute putative lignin binding faces, which may be illuminated in future studies. Similarly, residues putatively conferring preference for the backbone of glucuronoxylan poly- or oligosaccharides have here been identified but require validation in future studies.

Not only did some of the investigated bacterial CE15 members show unprecedented catalytic efficiencies on model substrates, but improved saccharification of unpretreated biomass was observed for GE-supplemented commercial lignocellulolytic enzyme cocktails. LCC-cleaving activities may be particularly important in the initial stages of hydrolysis of intact biomass, where the GEs likely increase the accessibility of other glycolytic enzymes to polysaccharides in the complex plant cell wall matrix. In addition, a tight functional relationship between GEs and xylanases can be postulated, where GEs act either prior to, or in concert with, *endo*-xylanases to detach xylan from lignin, or release xylanase-generated xylooligosaccharides from lignin networks for further degradation into monosaccharides. In agreement with our study, recent work has reported a synergistic cooperation of a fungal GE and xylanases [21]. However, we also detected striking increases in glucose concentration in GE-enhanced reactions, indicating that not only xylanases, but a range of lignocellulolytic enzymes are aided by the GE action. In-depth understanding of the enzymatic cleavage of LCCs in native structures and enzyme cooperativity may be revealed by future enzyme synergy studies on complex plant biomass.

## Conclusion

By structural and functional characterization, as well as gene expression analysis of a range of bacterial GEs spanning the CE15 phylogenetic tree, we have significantly expanded the existing knowledge on CE15 enzymes. Enzyme kinetic analyses of diverse bacterial CE15 members highlight common features as well as functional diversity. The boosting effects of bacterial GEs on biomass saccharification supports the proposed role of the enzymes to aid in reducing lignocellulose recalcitrance while variations in transcriptional responses of CE15-encoding genes during different growth conditions suggests nonredundant functions for enzymes found in species with multiple CE15 genes, possibly indicating that biomass specificity exists within the CE15 family. Taken together, the results provide a foundation for further fundamental and applied research regarding microbial degradation of recalcitrant plant cell walls.

## Methods

### Phylogenetic analysis

The protein sequences of all CE15 members (214 entries) were downloaded from CAZy (Feb 2018) and used to construct the phylogenetic tree as described previously [33]. Briefly, the sequences were trimmed to comprise only catalytic domains, aligned using MUSCLE [34], and the tree computed using PHYML [35].

### Cloning, expression, and purification of bacterial CE15 genes

The CE15 genes were amplified from genomic DNA of *O. terrae* DSM 11246, *S. linguale* DSM 74 and *S. usitatus* DSM 15142 (DSMZ, Germany) by PCR (primers in Additional file 1: Table S3), and the products cloned into modified pET-28a vectors (In-Fusion HD kit, Clontech Laboratories), containing N-terminal His_6_ tags and TEV protease cleavage sites (generously provided by N. Koropatkin, University of Michigan). The *Ot*CE15 genes, *Sl*CE15A, *Su*CE15A and *Su*CE15C were expressed in *E. coli* BL21(λDE3). *Ot*CE15D was expressed in *E. coli* Rosetta2(λDE3). *Sl*CE15B and *Sl*CE15C were coexpressed with translation elongation factor (tig) from pTf16 and *Su*CE15B with groES-groEL-tig from p6-Tf2 (Clontech Laboratories) to yield sufficient soluble protein.

Cells were grown in antibiotics-supplemented lysogeny broth (LB) at 37 °C and 200 rpm under shaking until attaining an OD_600_ ~ 0.5 when expression was induced at by addition of isopropyl β-d-1-thiogalactopyranoside (IPTG) to a final concentration of 0.2 mM and the cells incubated at 16 °C overnight. For confirming chaperone coexpression, chaperones were induced at an OD_600_ ~ 0.3 by addition of 1 mg/mL l-arabinose (pTf16) or 10 ng/mL tetracycline (p6-Tf2), followed by IPTG induction as described at OD_600_ ~ 0.5. Cells were harvested by centrifugation (5000×*g* 10 min), resuspended in 20 mM tris(hydroxymethyl)aminomethane (TRIS) buffer (pH 8) containing 250 mM NaCl, 5 μg/mL lysozyme, and 10 μg/mL DNase, and disrupted by sonication. Cell debris was removed by centrifugation (18,000×*g*, 10 min), and proteins were purified using immobilized metal ion affinity chromatography on an ÄKTA system (GE healthcare) using 5 mL HisTrap™ Excel columns, with 50 mM TRIS (pH 8), 250 mM NaCl as binding buffer, and one-step elution (binding buffer incl. 250 mM imidazole), followed by dialysis into 50 mM TRIS buffer (pH 8). *Ot*CE15A and *Su*CE15C were further purified by anion and cation exchange chromatography, respectively. Anion exchange was performed on a HiLoad™ 16/10 Q Sepharose column (GE healthcare) with 50 mM Tris (pH 8) as loading buffer and elution using a linear gradient to 1 M NaCl. Cation exchange was performed on a HiLoad™ 16/10 SP Sepharose column (GE healthcare) with 50 mM sodium acetate (pH 5) as loading buffer and elution using a linear gradient to 1 M NaCl.

### Enzyme assays

Esterase-mediated uronic acid formation was monitored continuously using the K-URONIC kit (Megazyme, Ireland). Kinetic measurements were performed in 96-well plates using a FLUOstar Omega (BMG LABTECH, Germany) in 200 μL reactions containing 50 mM sodium phosphate, 2 μL uronate dehydrogenase, and 16 μL NAD^+^. The buffer pH was at or close to the enzymes’ respective pH optima, due to substrate instability at higher pH, and where > 75% of maximal enzyme activity is maintained: pH 7.5 for *O. terrae* and *S. usitatus* enzymes and pH 6.5 for *S. linguale* enzymes. The substrates BnzGlcA, AllylGlcA, MeGlcA, and MeGalA (Additional file 1: Figure S2) (Carbosynth, UK) were dissolved in 100% dimethyl sulfoxide (DMSO); all reactions contained ≤ 10% DMSO. Kinetic assays were performed at least in duplicate at 25 °C using enough enzyme to ensure ≥ 2-fold change in substrate turnover versus auto-hydrolysis rates. pH-dependency profiles were generated with 2 mM BnzGlcA in a three-component buffer containing 25 mM acetic acid, 25 mM 2-(*N*-morpholino)ethanesulfonic acid, and 50 mM Tris–HCl, covering pH 4.5–9.5 [36].

Acetyl esterase activity was assayed using 4-nitrophenyl acetate (*p*NP-Ac; Sigma Aldrich) and 1,2,3,4-tetra-*O*-acetyl-β-d-xylopyranose (TetAcXyl; Carbosynth) (Additional file 1: Figure S2). *p*NP release was detected at λ_405_ and quantified using an extinction coefficient 18.7 mM^−1^cm^−1^. Acetate release from TetAcXyl were measured using the K-ACET kit (Megazyme). Pectin methyl esterase activity was assayed with poly-d-galacturonic acid methyl ester (Carbosynth) and citrus peel pectin (Sigma Aldrich) in reactions containing 0.2% (w/v) pectin and 1 mg/mL CE15 enzyme. Reactions were collected at 30 min and 24 h, filtered through a 10 kDa Amicon spin filter, and methanol release through NAD^+^ reduction using alcohol oxidase (*Pichia pastoris*, Sigma Aldrich) and formaldehyde dehydrogenase (*Pseudomonas* sp., Sigma) as previously described [37]. Nonlinear data were fitted to the Michaelis–Menten equation using GraphPad Prism (GraphPad, US). In nonsaturable cases, *k*_*cat*_/*K*_m_ values were determined by linear regression.

### Quantification of *S. linguale* gene expression by qPCR

*Spirosoma linguale* DSM 74 was grown in media containing 0.5 g/L peptone, 0.1 g/L yeast extract, 15 mM NaPO_4_ pH 7.5, 1 mM MgSO_4_, 2 mL/L trace metal solution [38] and 0.3% (w/v) of carbon source (glucose, xylose, corn cob xylan or ball milled corn cob). Milled corn cob was sterilized with 70% ethanol, dried, and washed with water to remove soluble sugars before use. 4 mL cultures, inoculated to a 1/100 dilution of an overgrown culture, were incubated at 30 °C with 200 rpm shaking. At mid-log phase [20 h for monosaccharides and xylan, and 36 h for biomass (Additional file 1: Figure S6)], cells were harvested by centrifugation and resuspended in 1 mL of TRIzol (Invitrogen). To the Trizol-resuspended cell pellets, 200 μL of chloroform was added, the reaction mixed, and the aqueous phase collected. RNA was precipitated in two volumes of isopropanol, washed twice with 500 μL 70% ethanol, and resuspended in 50 μL water. RNA samples were treated with Turbo DNase (Invitrogen), and cDNA was synthesized using RevertAid H Minus First Strand cDNA kit (ThermoFisher). qPCR was performed using primers, listed in Additional file 1: Table S3, and DyNAmo HS SYBR green (ThermoFisher) on a Stratagene MX3005P qPCR instrument (Agilent Technologies) using the following protocol: initial denaturation, 10 min at 95 °C, 40 cycles of 30-s denaturation at 95 °C, and 30-s annealing/elongation at 60 °C. Specificity of the amplicons was determined by DNA duplex dissociation by 1 min at 95 °C, 1 min at 55 °C, and 30 s at 95 °C. Three technical replicates of each biological triplicate were evaluated. Control reactions without a template gave no amplification, while controls with RNA instead of cDNA (RT-controls) had a *C*_t_ value > 5 cycles higher than the *C*_t_ value of the target reaction, indicating low background levels of genomic DNA. The MxPro software (Agilent Technologies) was used to analyze the data. The RNA polymerase sigma factor *rpoD* (Locus tag: Slin_1987) was used to normalize the data, based on predicted expression stability under the experimental conditions. Relative gene expression was quantified compared with growth on glucose using the 2^−ΔΔCt^ method [39].

### GE-aided corn cob saccharification

2 mL hydrolysis reactions containing 0.5% (w/v) ball milled corncob, 0.1 mg Ultraflo^®^ (Novozymes, Denmark)/g DW, without or supplemented with 175 nM CE15 enzyme (*Sl*CE15A, *Su*CE15A or *Su*CE15C) were performed in triplicate experiments, in 50 mM sodium acetate (pH5.5) at 35 °C with 1000 rpm mixing. Reactions were stopped after 10 min by heating at 95 °C. A low concentration of Ultraflo^®^ was chosen with to obtain limiting enzymatic hydrolysis conditions. No BnzGlcA-cleaving activity was detected in Ultraflo^®^. Released monosaccharides were monitored by high-performance anion exchange chromatography with pulsed amperometric detection on an ICS3000 system equipped with a 4 × 250 mm Dionex Carbopac™ PA1 column with a 4 × 50 mm guard column maintained at 30 °C, (Dionex, Sunnyvale, CA, USA). 25 μL samples were injected. The eluents were—A: Water; B: 300 mM sodium hydroxide, and C: 100 mM sodium hydroxide and 85 mM sodium acetate. The samples were eluted isocratically with 100% eluent A for 40 min (1 mL/min) and detected with postcolumn addition of 0.5 mL/min of solvent B. Thereafter, a cleaning step with 40% eluent A and 60% eluent B was performed at 1 mL/min for 10 min. Peak analysis was performed using the Chromeleon software. Peaks were quantified against pure monosaccharide standards, and 10 mg/L fructose was added as an internal standard.

### Crystallization and data collection

Tag-free *Ot*CE15A and *Su*CE15C, generated by His-tag cleavage by TEV protease and cleaned by passing through a 5 mL HisTrap™ Excel column, were screened for crystallization in MRC 2-drop crystallization plates (Molecular Dimensions) using an Oryx 8 Robot (Douglas Instrument). Sitting drops (0.3 µL) were mixed with protein:reservoir volume ratios of 3:1 or 1:1 using 45 mg/mL of *Ot*CE15A or 20 mg/mL of *Su*CE15C, both in 20 mM TRIS pH 8.0. Hits from Morpheus screens (Molecular Dimensions) were optimized, and final crystallization conditions were as follows: 0.09M NPS, 0.1 M Buffer system 3, and 37.5% v/v Precipitant mix 4 for *Ot*CE15A; 0.12 M Ethylene glycols, 0.1 M Buffer System 3, and 50% v/v Precipitant Mix 1 for *Su*CE15C [40]. Datasets were collected on ID30B at the ESRF, Grenoble, France, and crystals of both proteins diffracted beyond 2 Å. The initial *Ot*CE15A dataset collected belonged to space group P1 and diffracted to a resolution of 1.34 Å but had limited completeness (88.9%). Molecular replacement using the previously determined fungal CE15 structures as templates (25–30% identity) was unsuccessful in each case, and collection of anomalous data was pursued. The pET-28a constructs were transformed into *E. coli* T7 Express Crystal (methionine auxotroph; NEB), and the protein was expressed in minimal media containing seleno-l-methionine (SeMet) as per the supplier’s recommendations (Molecular Dimensions). SeMet CE15 proteins, without the N-terminal His-tag removed, were screened for crystallization conditions, and only the *Su*CE15C SeMet-substituted protein yielded well-diffracting crystals. Crystals of the *Su*CE15C-SeMet were grown in 0.12 M Monosaccharides, 0.1 M Buffer System 3, 50% v/v Precipitant Mix 1 [40]. Heavy-atom derivatization was trialed with native crystals of *Ot*CE15A, and the final conditions achieved were obtained by adding 1.2 μL of crystallization mother liquor containing 0.5 mM of KAu(CN)_2_ to the drop containing crystals. Crystals of the *Ot*CE15A used for derivatization were grown in 0.06 M Divalents, 0.1 M Buffer system 1, 40% v/v Precipitant mix 4 [40]. Derivatized crystals were soaked for 1 h before being flash-frozen in liquid nitrogen. Datasets of both the *Su*CE15A-SeMet and gold-derivatized *Ot*CE15A were collected at beamline P11 of Petra III.

### Data processing and structure determination

Diffraction data were processed with XDS [41] and structure solution completed in Phenix [42]. Autosol was used to solve the structure of the *Su*CE15C-SeMet which was subsequently used as a template for molecular replacement in Phaser to solve the native *Su*CE15C structure [42, 43]. Due to the better overall completeness, the *Ot*CE15A structure was first determined using the gold-derivatized dataset, by molecular replacement in Phaser using the *Su*CE15C as a template, and the model was subsequently used as a template for molecular replacement in Phaser to solve the native structure [42, 43]. Models of all the structures were initially built with Phenix AutoBuild [44], rebuilt in Coot [45], and further refined with Phenix Refine [46] in alternating cycles. OtCE15A structures were refined isotropically after checking that an anisotropic refinement scheme did not bring any significant improvement. Ligand compounds were added to the models in Coot, and the identity of the metals in the *Ot*CE15A structure was validated using CheckMyMetal server [47]. Due to disorder and poor density, a small loop in the *Ot*CE15A-Au (residues 221–223), two N-terminal residues in the *Ot*CE15A-Native (Ala33 and Tyr34), the C-terminal residue in *Ot*CE15A-Au (Ala432), one residue in the *Su*CE15C-Native structure (Asp346), and one residue’s side chain in the *Su*CE15C-SeMet (Trp327) were unable to be confidently modeled and were omitted. One loop in the *Ot*CE15A-Native structure (residues 423–429) was modeled in double conformation. All models had ≥ 96% residues’ in the most favorable Ramachandran regions [48]. Additional file 1: Table S4 lists the data collection and final model refinement statistics.

### Alignments and docking simulations

The multiple sequence alignment was completed with Clustal Omega [49] and the structural alignment was completed with DALI [50]. Both alignments were visualized with ESPRIPT [51] using the default percent equivalent coloring scheme. Docking simulations were completed with ROSIE using the atomic coordinates of *Ot*CE15A, *Su*CE15C, and *St*GE2 (PDB accession: 4g4g) as the templates [52–55]. The 4-*O*-methyl-glucuronoxylotriose (glucuronoate α-1,2 linked to the middle xylose residue) ligand was created in MarvinSketch (ChemAxon) and parameters in ROSIE were set to generate 500 different ligand conformers. A 5 Å search radius from center of the pocket, defined by equivalent positions to the center of the pyranose ring found in *St*GE2 (PDB accession: 4g4j), was utilized and over 1000 docking poses were generated. All other search parameters were set to default conditions. The top 300 poses containing the lowest interface delta score were chosen for further analyses.

## Additional file

**Additional file 1: Table S1.** Percent sequence identity and percent query coverage (in brackets) between all CE15 enzymes used in this study. Sequence identity values for CE15 enzymes within one organism are marked green (*O. terrae*), magenta (*S. linguale*) and blue (*S. usitatus*). The query sequences are presented in the top row.Kinetic parameters of *O. terrae*, *S. linguale*, and *S. usitatus* CE15 enzymes on model. **Table S2.** Kinetic parameters of *O. terrae*, *S. linguale*, and *S. usitatus* CE15 enzymes on model substrates. Esterase activity with benzyl (Bnz), allyl (Allyl), methyl (Me) esters of glucuronoate (GlcA) and galacturonoate (GalA) are shown in addition to acetyl esterase activity with 4-nitrophenol acetate (*p*NP-Ac) and 1,2,3,4-tetra-*O*-acetyl-β-d-xylopyranose (TetAcXyl). **Table S3.** Primers used for cloning CE15 constructs and for qPCR of S. linguale CE15 members. **Table S4.** Table of crystallographic statistics. **Figure S1.** Unrooted phylogenetic tree of all members of CE15 (catalytic domains), with Genbank accession numbers as identifiers. Yellow branches represent fungal members, circles indicate biochemically characterized members, and stars represent members with solved structures. Targets of this study are shown using the same color code as in the main text: green for *O. terrae*, red for *S. linguale*, and blue for *S. usitatus*. **Figure S2.** Model substrates used in this study: (A) BnzGlcA, (B) AllylGlcA, (C) MeGlcA, (D) MeGalA, (E) pNP-Ac and (F) TetAcXyl. **Figure S3.** Effect of pH on BnzGlcA esterase activity for CE15 enzymes from *O. terrae* (OtCE15 A-D, panels A-D), *S. linguale* (SlCE15 A-C, panels E-G), and *S. usitatus* (SuCE15 A-C, panels H-J). Mean values of relative activity from duplicate measurements are plotted with standard error of the mean. **Figure S4.** Structure-based sequence alignment of all CE15 enzymes structurally characterized to date. Similar residues are written in red text while conserved residues are written in white text over a red background. The insertion regions found in the bacterial structures relative to the fungal counterparts are highlighted in yellow. The residues of the canonical catalytic triad are indicated by cyan arrows below the text. The aspartate in MZ0003 proposed to act as the acidic residue of the catalytic triad, in place of the missing canonical glutamate, is indicate by a black arrow below the text. Note that both *Ot*CE15A and *Su*CE15C also have an aspartate at the same position while additionally having the glutamate of the canonical catalytic triad. Residues hydrogen bonding with 4-O-methyl-glucuronoate in the *St*GE2 co-crystal structure are indicated by blue arrows above the text. The isoleucine and leucine comprising a hydrophobic patch near the 4-O-methyl substituent in the *St*GE2 co-crystal structure are indicated by magenta arrows. The phenylalanine conserved in the bacterial structures possibly aiding in positioning in aromatic substituents of the sugar esters is indicated with a grey arrow. The disulfide bridges formed in the fungal structures are indicated above the alignment by numbering in green text. **Figure S5.** Multiple sequence alignment of characterized glucuronoyl esterases. Similar residues are written in red text while conserved residues are written in white text over a red background. The insertion regions found in the bacterial structures relative to the fungal counterparts are highlighted in yellow. The residues of the conserved catalytic triad are colored cyan. Note that glutamate of the catalytic triad is not conserved in all bacterial esterases and the position of the equivalent acidic residue in MZ0003 is also colored cyan. Arrows indicating significant residues are colored as in Additional file 5: Figure S4. **Figure S6.** Growth curves of *S. linguale* when grown with different additives or on different carbon sources. *S. linguale* did not grow on standard minimal media and an optimized media for bacterial growth was determined experimentally (see methods for formulation). (A) Growth curves of *S. linguale* in the optimized media without a carbon source (red), with 0.3% (w/v) glucose (blue) and in the media containing glucose but in the absence of either trace metals and vitamins (green), sodium phosphate pH 7.5 (magenta), or magnesium sulphate (cyan). (B) Growth of *S. linguale* in optimized media with 0.3% of either glucose (blue), xylose (purple), or xylan from corn cob (yellow).

## Abbreviations

GE: glucuronoyl esterase
CE15: carbohydrate esterase 15
LCC: lignin carbohydrate complex
CAZy: carbohydrate active enzyme database
GlcA: glucuronoate/glucuronic acid
GalA: galacturonoate/galacturonic acid
BnzGlcA: benzyl glucuronoate
AllylGlcA: allyl glucuronoate
MeGlcA: methyl glucuronoate
MeGalA: methyl galacturonoate
*p*NP-Ac: 4-nitrophenol acetate
PDB: protein data bank
IPTG: isopropyl β-d-1-thiogalactopyranoside
