# Supplementary material for: Biochemical and structural features of diverse bacterial glucuronoyl esterases facilitating recalcitrant biomass conversion
Source: Biotechnol Biofuels. 2018 Aug 1;11:213. doi: 10.1186/s13068-018-1213-x (PMC6069808; doi:10.1186/s13068-018-1213-x)
Supplement: Supplementary file 1 — Additional file 1: Table S1. Percent sequence identity and percent query coverage (in brackets) between all CE15 enzymes used in this study. Sequence identity values for CE15 enzymes within one organism are marked green (O. terrae), magenta (S. linguale) and blue (S. usitatus). The query sequences are presented in the top row.Kinetic parameters of O. terrae, S. linguale, and S. usitatus CE15 enzymes on model. Table S2. Kinetic parameters of O. terrae, S. linguale, and S. usitatus CE15 enzymes on model substrates. Esterase activity with benzyl (Bnz), allyl (Allyl), methyl (Me) esters of glucuronoate (GlcA) and galacturonoate (GalA) are shown in addition to acetyl esterase activity with 4-nitrophenol acetate (pNP-Ac) and 1,2,3,4-tetra-O-acetyl-β-d-xylopyranose (TetAcXyl). Table S3. Primers used for cloning CE15 constructs and for qPCR of S. linguale CE15 members. Table S4. Table of crystallographic statistics. Figure S1. Unrooted phylogenetic tree of all members of CE15 (catalytic domains), with Genbank accession numbers as identifiers. Yellow branches represent fungal members, circles indicate biochemically characterized members, and stars represent members with solved structures. Targets of this study are shown using the same color code as in the main text: green for O. terrae, red for S. linguale, and blue for S. usitatus. Figure S2. Model substrates used in this study: (A) BnzGlcA, (B) AllylGlcA, (C) MeGlcA, (D) MeGalA, (E) pNP-Ac and (F) TetAcXyl. Figure S3. Effect of pH on BnzGlcA esterase activity for CE15 enzymes from O. terrae (OtCE15 A-D, panels A-D), S. linguale (SlCE15 A-C, panels E-G), and S. usitatus (SuCE15 A-C, panels H-J). Mean values of relative activity from duplicate measurements are plotted with standard error of the mean. Figure S4. Structure-based sequence alignment of all CE15 enzymes structurally characterized to date. Similar residues are written in red text while conserved residues are written in white text over a red background. The inserti [file 13068_2018_1213_MOESM1_ESM.docx]

**Table S1.** Percent sequence identity and percent query coverage (in brackets) between all CE15 enzymes used in this study. Sequence identity values for CE15 enzymes within one organism are marked green (*O. terrae*), magenta (*S. linguale*) and blue (*S. usitatus*). The query sequences are presented in the top row. The percentages were calculated using BLAST (https://blast.ncbi.nlm.nih.gov/Blast.cgi).

|  | *Ot*A | *Ot*B | *Ot*C | *Ot*D | *Sl*A | *Sl*B | *Sl*C | *Su*A | *Su*B | *Su*C |
| --- | --- | --- | --- | --- | --- | --- | --- | --- | --- | --- |
| *Ot*A | **100** | 27(52) | 43(62) | 39(73) | 50(98) | 34(74) | 34(81) | 44(96) | 33(57) | 48(98) |
| *Ot*B | 27(78) | **100** | 26(36) | 29(71) | 35(41) | 26(64) | 29(70) | 27(95) | 25(50) | 30(39) |
| *Ot*C | 43(90) | 26(54) | **100** | 43(73) | 42(90) | 38(86) | 32(85) | 58(98) | 33(70) | 46(94) |
| *Ot*D | 39(89) | 30(67) | 43(61) | **100** | 37(92) | 48(99) | 42(86) | 39(98) | 47(58) | 38(92) |
| *Sl*A | 51(91) | 35(30) | 42(61) | 37(77) | **100** | 34(75) | 33(78) | 44(93) | 33(59) | 53(92) |
| *Sl*B | 34(90) | 26(52) | 38(62) | 48(97) | 34(92) | **100** | 37(85) | 38(95) | 44(58) | 36(93) |
| *Sl*C | 34(79) | 29(54) | 32(62) | 42(76) | 33(88) | 37(83) | **100** | 32(92) | 60(90) | 34(87) |
| *Su*A | 44(92) | 27(64) | 58(72) | 39(86) | 42(98) | 38(76) | 32(83) | **100** | 34(61) | 50(93) |
| *Su*B | 33(91) | 25(54) | 32(71) | 47(83) | 33(98) | 44(76) | 57(94) | 32(94) | **100** | 35(83) |
| *Su*C | 47(96) | 26(26) | 46(63) | 38(76) | 53(92) | 36(75) | 41(58) | 49(94) | 35(60) | **100** |

**Table S2.** Kinetic parameters of *O. terrae*, *S. linguale, and S. usitatus* CE15 enzymes on model substrates. Esterase activity with benzyl (Bnz), allyl (Allyl), methyl (Me) esters of glucuronoate (GlcA) and galacturonoate (GalA) are shown in addition to acetyl esterase activity with 4-nitrophenol acetate (*p*NP-Ac) and 1,2,3,4-tetra-*O*-acetyl-β-d-xylopyranose (TetAcXyl).

| **Enzyme** | **Substrate** | ***K_m_* (mM)** | ***k_cat_* (s^-1^)** | | | | ***k_cat_/K_m_* (s^-1^M^-1^)** |
| --- | --- | --- | --- | --- | --- | --- | --- |
| *Ot*CE15A | BnzGlcA | 4.18 ± 0.14 | 19.4 ± 0.19 | | | | (4.64 ± 0.16) x 10^3^ |
|  | AllylGlcA | 2.87 ± 0.10 | 25.2 ± 0.22 | | | | (8.80 ± 0.31) x 10^3^ |
|  | MeGlcA | 2.77 ± 0.15 | 19.0 ± 0.31 | | | | (6.85 ± 0.39) x 10^3^ |
|  | MeGalA | 5.31 ± 0.51 | 28.8 ± 0.64 | | | | (4.85 ± 0.47) x 10^3^ |
|  | *p*NP-Ac | *Cannot be saturated up to 10 mM* | | | | | (3.23 ± 0.063) x 10^1^ |
|  | TetAcXyl | *Cannot be saturated up to 10 mM* | | | | | (8.59 ± 0.65) x 10^-7^ |
| *Ot*CE15B | BnzGlcA | 7.94 ± 0.87 | 0.15 ± 0.0049 | | | | (1.86 ± 0.21) x 10^1^ |
|  | AllylGlcA | 12.5 ± 1.3 | 0.0354 ± 0.0015 | | | | 2.82 ± 0.32 |
|  | MeGlcA | 11.1 ± 2.3 | 0.0127 ± 0.00087 | | | | 1.14 ± 0.25 |
|  | MeGalA | 6.32 ± 0.59 | 0.055 ± 0.0014 | | | | 8.68 ± 0.84 |
|  | *p*NP-Ac | *Cannot be saturated up to 10 mM* | | | | | 2.56 ± 0.061 |
|  | TetAcXyl | *Cannot be saturated up to 10 mM* | | | | | (6.95 ± 0.38) x 10^-7^ |
| *Ot*CE15C | BnzGlcA | 0.449 ± 0.054 | 5.21 ± 0.14 | | | | (1.16 ± 0.14) x 10^4^ |
|  | AllylGlcA | 2.21 ± 0.12 | 5.48 ± 0.072 | | | | (2.49 ± 0.14) x 10^3^ |
|  | MeGlcA | 4.01 ± 0.055 | 3.60 ± 0.16 | | | | (8.98 ± 0.42) x 10^2^ |
|  | MeGalA | 1.93 ± 0.15 | 2.29 ± 0.039 | | | | (1.19 ± 0.094) x 10^3^ |
|  | *p*NP-Ac | *Cannot be saturated up to 10 mM* | | | | | (3.97 ± 0.070) x 10^1^ |
|  | TetAcXyl | 1.65 ± 0.16 | | 0.0132 ± 0.00039 | | | 8.04 ± 0.81 |
| *Ot*CE15D | BnzGlcA | 0.608 ± 0.024 | 6.24 ± 0.061 | | | | (1.11 ± 0.044) x 10^4^ |
|  | AllylGlcA | 2.08 ± 0.090 | 7.19 ± 0.072 | | | | (3.45 ± 0.15) x 10^3^ |
|  | MeGlcA | 12.3 ± 0.71 | 6.37 ± 0.13 | | | | (5.19 ± 0.32) x 10^2^ |
|  | MeGalA | *Cannot be saturated up to 50 mM* | | | | | (1.95 ± 0.030) x 10^-6^ |
|  | *p*NP-Ac | *Cannot be saturated up to 10 mM* | | | | | 9.51 ± 0.18 |
|  | TetAcXyl | 6.84 ± 1.2 | | 0.0240 ± 0.0022 | | | 3.51 ± 0.70 |
| *Sl*CE15A | BnzGlcA | 6.02 ± 0.36 | | 11.3 ± 0.23 | | | (1.88 ± 0.12) x 10^3^ |
|  | AllylGlcA | 10.3 ± 1.5 | | 10.3 ± 0.65 | | | (1.00 ± 0.16) x 10^3^ |
|  | MeGlcA | 4.99 ± 0.16 | | 7.75 ± 0.082 | | | (1.55 ± 0.051) x 10^3^ |
|  | MeGalA | 23.4 ± 1.6 | | 0.89 ± 0.029 | | | (3.82 ± 0.29) x 10^1^ |
|  | *p*NP-Ac | *Cannot be saturated up to 10 mM* | | | | | (3.09 ± 0.15) x 10^1^ |
| *Sl*CE15B | BnzGlcA | 0.623 ± 0.084 | | 1.62 ± 0.050 | | | (2.60 ± 3.6) x 10^3^ |
|  | AllylGlcA | 2.07 ± 0.093 | | 1.88 ± 0.024 | | | (9.08 ± 4.3) x 10^2^ |
|  | MeGlcA | 4.64 ± 0.087 | | 2.12 ± 0.013 | | | (4.57 ± 0.091) x 10^2^ |
|  | MeGalA | *Cannot be saturated up to 50 mM* | | | | | (3.66 ± 0.11) x 10^-7^ |
|  | *p*NP-Ac | *No Activity Detected above 0.5 µmol ∙ s^-1^ ∙ µmol_enzyme_^-1^ at 10 mM* | | | | | |
| *Sl*CE15C | BnzGlcA | 7.62 ± 0.56 | | 0.74 ± 0.023 | | | (9.69 ± 0.77) x 10^1^ |
|  | AllylGlcA | 10.4 ± 0.76 | | 1.15 ± 0.037 | | | (1.11 ± 0.088) x 10^2^ |
|  | MeGlcA | 8.61 ± 1.6 | | 0.883 ± 0.064 | | | (1.03 ± 0.20) x 10^2^ |
|  | MeGalA | *Cannot be saturated up to 50 mM* | | | | | (3.73 ± 0.13) x 10^-6^ |
|  | *p*NP-Ac | *No Activity Detected above 0.5 µmol ∙ s^-1^ ∙ µmol_enzyme_^-1^ at 10 mM* | | | | | |
| *Su*CE15A | BnzGlcA | 0.42 ± 0.080 | | | 9.25 ± 0.39 | (2.20 ± 0.43) x 10^4^ | |
|  | AllylGlcA | 1.24 ± 0.17 | | | 6.78 ± 0.23 | (5.47 ± 0.77) x 10^3^ | |
|  | MeGlcA | 4.31 ± 0.40 | | | 10.0 ± 0.26 | (2.32 ± 0.22) x 10^3^ | |
|  | MeGalA | 2.00 ± 0.25 | | | 3.24 ± 0.10 | (1.62 ± 0.21) x 10^3^ | |
|  | *p*NP-Ac | *Cannot be saturated up to 10 mM* | | | | 5.09 ± 0.32 | |
| *Su*CE15B | BnzGlcA | 12.6 ± 1.1 | | | 18.8 ± 0.84 | (1.49 ± 0.15) x 10^3^ | |
|  | AllylGlcA | 40.6 ± 1.4 | | | 14.8 ± 0.29 | (3.65 ± 0.15) x 10^2^ | |
|  | MeGlcA | *Cannot be saturated up to 25 mM* | | | | (6.00 ± 0.30) x 10^-2^ | |
|  | MeGalA | *Cannot be saturated up to 25 mM* | | | | (9.00 ± 0.10) x 10^-3^ | |
|  | *p*NP-Ac | *Cannot be saturated up to 10 mM* | | | | (1.82 ± 0.12) x 10^1^ | |
| *Su*CE15C | BnzGlcA | 2.55 ± 0.25 | | | 58.0 ± 1.7 | (2.27 ± 0.23) x 10^4^ | |
|  | AllylGlcA | 2.82 ± 0.13 | | | 44.3 ± 0.62 | (1.57 ± 0.076) x 10^4^ | |
|  | MeGlcA | 3.62 ± 0.33 | | | 60.1 ± 1.5 | (1.66 ± 0.16) x 10^4^ | |
|  | MeGalA | 15.0 ± 0.96 | | | 23.8 ± 0.61 | (1.59 ± 0.11) x 10^3^ | |
|  | *p*NP-Ac | *Cannot be saturated up to 10 mM* | | | | (1.09 ± 0.053) x 10^1^ | |

**Table S3.** Primers used for cloning of CE15 constructs and for qPCR of *S. linguale* CE15 members.

| **Gene** | **Primer** | **5’-3’ sequence** |
| --- | --- | --- |
| *Ot*CE15A | OtCE15Af  OtCE15Ar | TGGTGGTGCTCGAGTCTAAGCAGGCAGAGCCGATTTCAA  GCCGGCGATGGCCATGGCCTACACGTTACCGGATCCG |
| *Ot*CE15B | OtCE15Bf  OtCE15Br | CTTCCAGGGCCATAGTCACTCGGGTCGTCAGGATG  TGGTGGTGCTCGAGTCTAAGGTAGCCCGTGACCCC |
| *Ot*CE15C | OtCE15Cf  OtCE15Cr | CTTCCAGGGCCATAGTTCCAACGAAGCGCTAACCG  TGGTGGTGCTCGAGTCTAAGAGAGATGTTCCCGAACGAAGTC |
| *Ot*CE15D | OtCE15Df  OtCE15Dr | CTTCCAGGGCCATAGTGCTGAAAAACCTGCTCGGGC  TGGTGGTGCTCGAGTCTAGCCCAAATACCGGTCGGCAA |
| *Sl*CE15A | SlCE15Af  SlCE15Ar | CTTCCAGGGCCATAGTGGTCCAGAAGGCTATAACTACGACG  TGGTGGTGCTCGAGTCTAGCGAACTAATTCATCGGCGAAGC |
| *Sl*CE15B | SlCE15Bf  SlCE15Br | CTTCCAGGGCCATAGTGCGCCCGACTTTCAGAAAATGAC  TGGTGGTGCTCGAGTCTATTTCAACTGCAACTCTCGCTTC |
| *Sl*CE15C | SlCE15Cf  SlCE15Cr | CTTCCAGGGCCATAGTCAAACTGCAATCGACAGTAGCA  TGGTGGTGCTCGAGTCTACGAGAACAGCTTGCTAGCCCAG |
| *Su*CE15A | SuCE15Af  SuCE15Ar | ACTTCCAGGGCCATATGGCAGATAAACCGCCAGCCGCCGTC  GGTGGTGGTGCTCGAGTCAAGGCTTCAGGTGCATGCGGAGGAAGGC |
| *Su*CE15B | SuCE15Bf  SuCE15Br | ACTTCCAGGGCCATATGATGAACTGGACCGCCGCCGAG  GGTGGTGGTGCTCGAGGCCTAGGAGTTCGCGGAAGATAGGCTT |
| *Su*CE15C | SuCE15Cf  SuCE15Cr | ACTTCCAGGGCCATATGATCACGGACGAGGCGAAAGTTCCTGCC  GGTGGTGCTCGAGTCAGCCCCATTGTGCTTTTGCGAACTTCAGGTACTG |
| *slce15A* | *slce15A*-f  *slce15A*-r | GCTGGGTACAACGGTCTTTCC  TCGTAGTCCGTTACATCATGTTTGC |
| *slce15B* | *slce15B*-f *slce15B*-r | GGAAAGCTAAATCTTCCGCTGC  AGTTGCTGGAACCTTCCTTCTG |
| *slce15C* | *slce15C*-f  *slce15C*-r | GCAACGGCAAGAAAGTAACCAC  TAGTTTACCATACATTTCCCGCTCG |
| *rpoD* | *rpoD*-f  *rpoD*-r | ACCAATTGTCGCTGGAACGATTAA  CAGGTCACCTAATGAAAGCCCC |

**Table S4:** Table of crystallographic statistics.

|  | *Su*CE15C-SeMet | *Su*CE15C-Native | *Ot*CE15A-Au | *Ot*CE15A-Native |
| --- | --- | --- | --- | --- |
| Data Collection |  |  |  |  |
| Date | July 26, 2017 | April 27, 2017 | July 26, 2017 | April 27, 2017 |
| Source | P11 at Petra III | ID30B at ESRF | P11 at Petra III | ID30B at ESRF |
| Wavelength (Å) | 0.9795 | 0.8999 | 0.9795 | 0.8999 |
| Space group | P4_3_2_1_2 | P4_3_2_1_2 | P1 | P1 |
| Cell dimensions |  |  |  |  |
| *a, b, c* (Å) | 54.58, 54.58, 284.11 | 54.58, 54.58, 284.11 | 43.69, 44.61, 50.56 | 43.36, 44.26, 51.39 |
| α, β, γ (°) | 90, 90, 90 | 90, 90, 90 | 76.57, 67.17, 70.69 | 87.79, 116.93, 109.12 |
| No. of measured reflections | 59163 (5723) | 245204 (22245) | 180550 (28703) | 188328 (13846) |
| No. of independent reflections | 29591 (2871) | 29889 (2515) | 50802 (4917) | 63925 (4728) |
| Resolution (Å) | 43.28 - 2.02 (2.09 - 2.02) | 43.28 - 2.00 (2.07 - 2.00) | 46.27 - 1.50 (1.55 - 1.50) | 45.43-1.34 (1.37 - 1.34) |
| *R*_merge_ (%) | 6.91 (42.61) | 11.57 (90.87) | 4.2 (31.4) | 9.2 (107.3) |
| CC_1/2_ | 99.9 (88.9) | 99.8 (49.0) | 99.9 (92.3) | 99.5 (53.2) |
| Mean I/σI | 9.27 (1.35) | 11.58 (2.02) | 16.69 (3.40) | 6.35 (0.97) |
| Completeness | 99.95 (99.65) | 98.42 (85.34) | 96.10 (92.93) | 88.9 (88.6) |
| Redundancy | 2.0 (2.0) | 8.1 (7.6) | 3.6 (3.6) | 2.9 (2.9) |
| Refinement |  |  |  |  |
| *R*_work_/*R*_free_ | 0.184/0.249 | 0.188/0.239 | 0.135/0.160 | 0.173/0.207 |
| No. atoms |  |  |  |  |
| Protein | 3118 | 3097 | 3167 | 3201 |
| Ligand/ions | 53 | 130 | 77 | 120 |
| Water | 277 | 222 | 402 | 374 |
| B-factors |  |  |  |  |
| Protein | 40.51 | 38.22 | 23.25 | 21.80 |
| Ligand/ions | 62.62 | 61.94 | 38.60 | 39.49 |
| Water | 44.80 | 42.43 | 34.69 | 29.68 |
| RMSD |  |  |  |  |
| Bond length (Å) | 0.012 | 0.013 | 0.009 | 0.009 |
| Bond angles (°) | 1.15 | 1.15 | 1.38 | 1.33 |
| PDB accession | 6GU8 | 6GRY | 6GRW | 6GS0 |


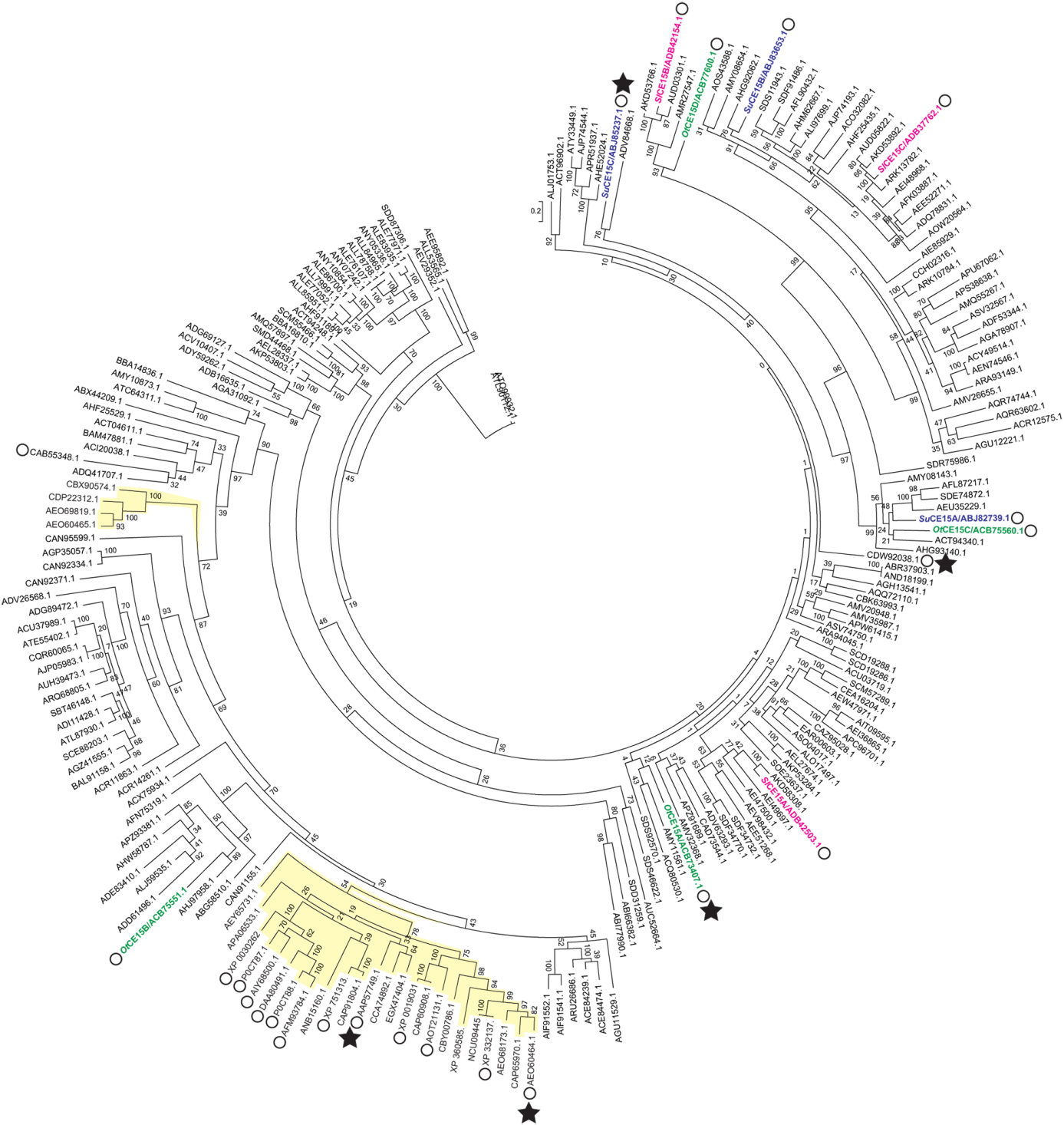


**Figure S1.** Unrooted phylogenetic tree of all members of CE15 (catalytic domains), with Genbank accession numbers as identifiers. Yellow branches represent fungal members, circles indicate biochemically characterized members, and stars represent members with solved structures. Targets of this study are shown using the same color code as in the main text: green for *O. terrae*, magenta for *S. linguale*, and blue for *S. usitatus*.


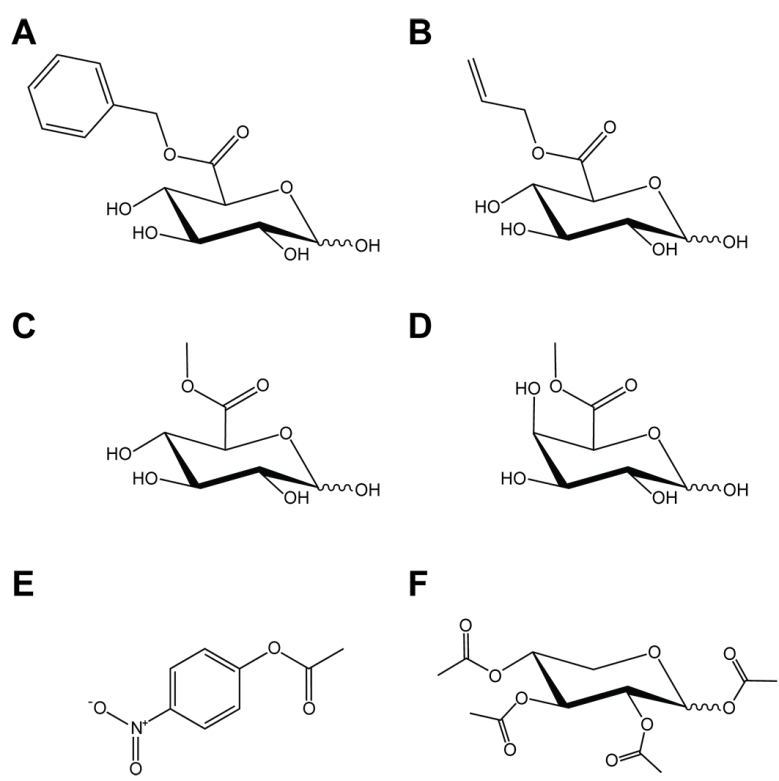


**Figure S2.** Model substrates used in this study: (A) BnzGlcA, (B) AllylGlcA, (C) MeGlcA, (D) MeGalA, (E) *p*NP-Ac and (F) TetAcXyl.


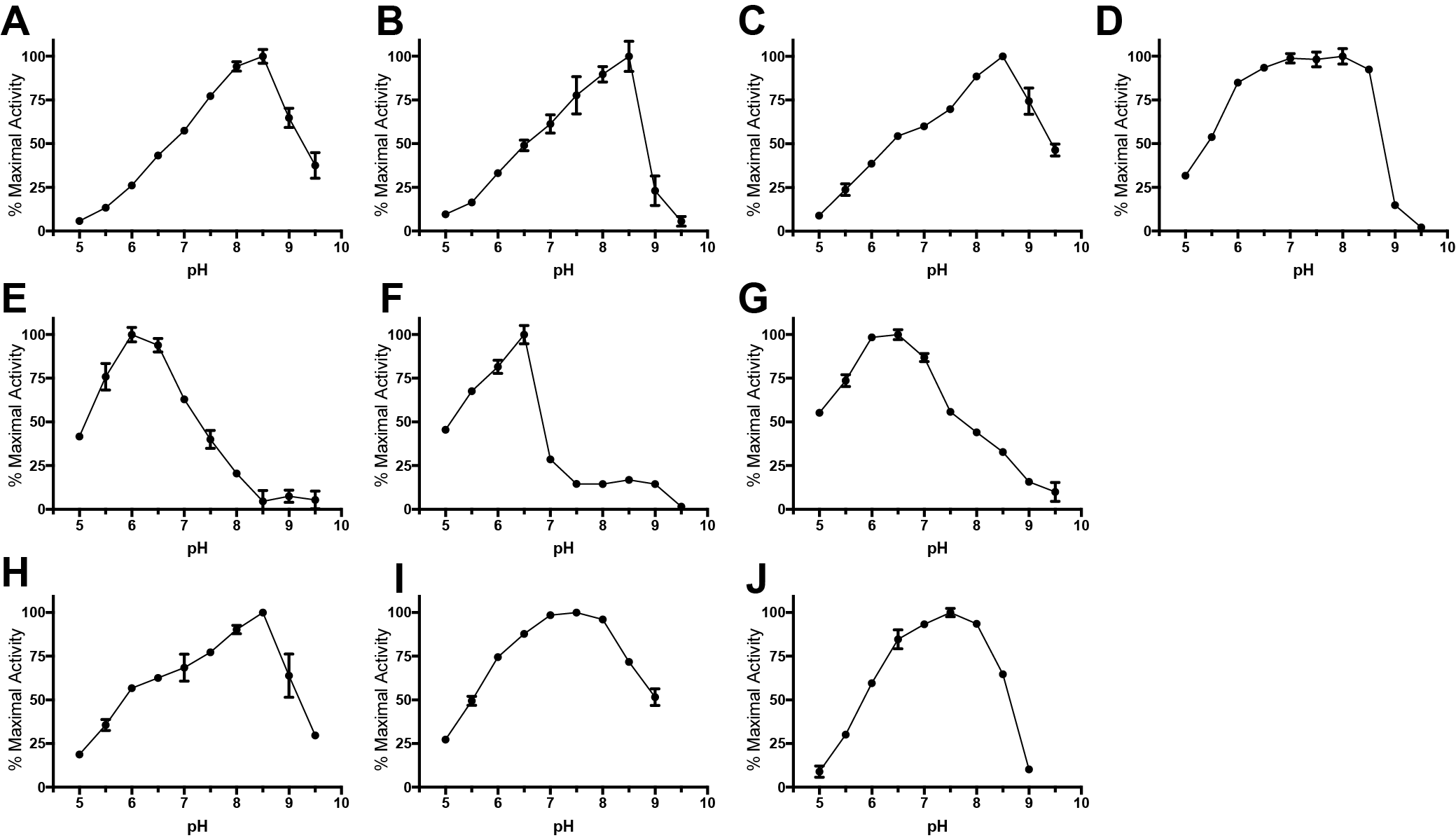


**Figure S3.** Effect of pH on BnzGlcA esterase activity for CE15 enzymes from *O. terrae* (*Ot*CE15 A-D, panels A-D), *S. linguale* (*Sl*CE15 A-C, panels E-G), and *S. usitatus* (*Su*CE15 A-C, panels H-J). Mean values of relative activity from duplicate measurements are plotted with standard error of the mean.


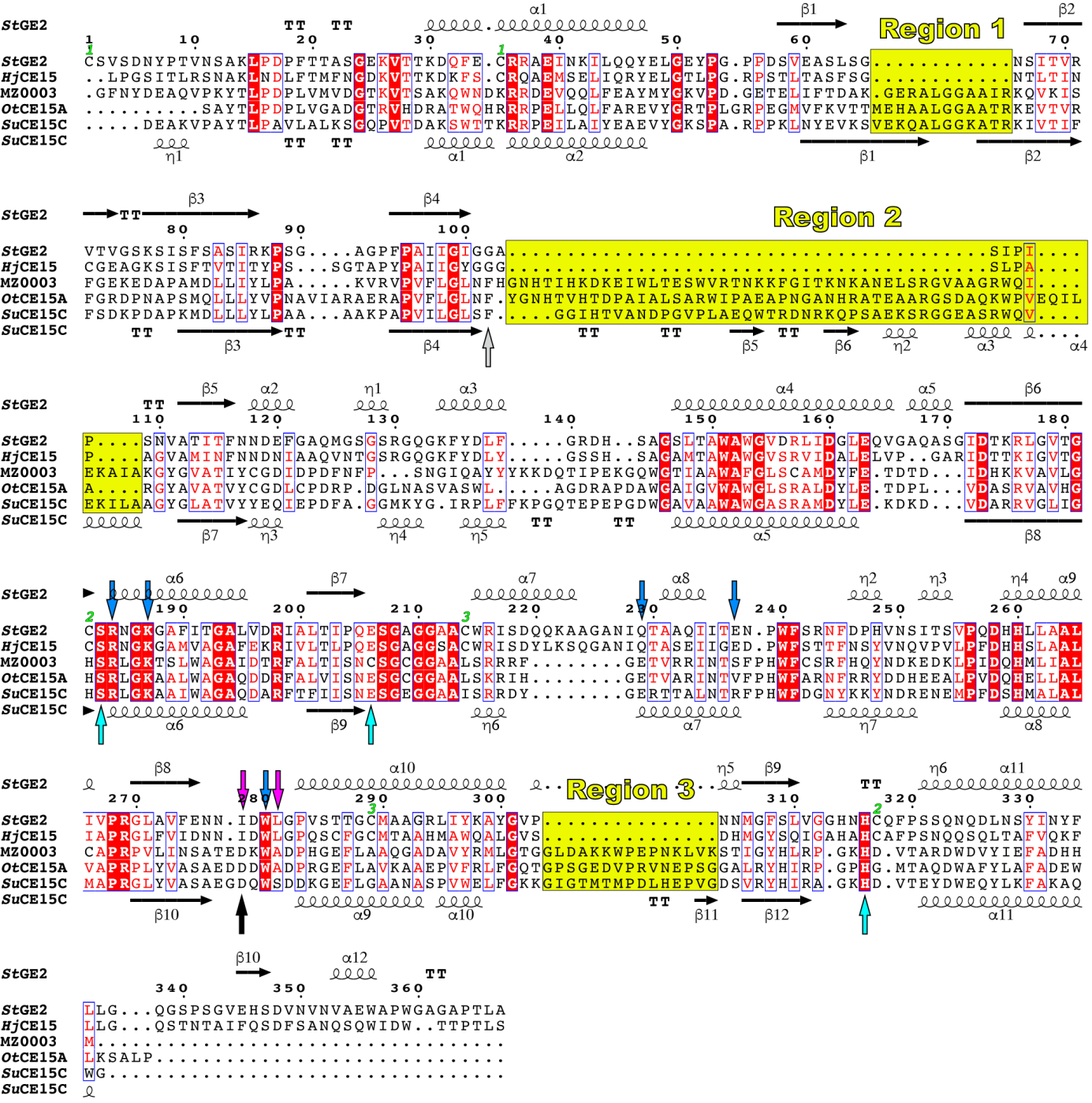


**Figure S4.** Structure-based sequence alignment of all CE15 enzymes structurally characterized to date. Similar residues are written in red text while conserved residues are written in white text over a red background. The insertion regions found in the bacterial structures relative to the fungal counterparts are highlighted in yellow. The residues of the canonical catalytic triad are indicated by cyan arrows below the text. The aspartate in MZ0003 proposed to act as the acidic residue of the catalytic triad, in place of the missing canonical glutamate, is indicate by a black arrow below the text. Note that both *Ot*CE15A and *Su*CE15C also have an aspartate at the same position while additionally having the glutamate of the canonical catalytic triad. Residues hydrogen bonding with 4-*O*-methyl-glucuronoate in the *St*GE2 co-crystal structure are indicated by blue arrows above the text. The isoleucine and leucine comprising a hydrophobic patch near the 4-*O*-methyl substituent in the *St*GE2 co-crystal structure are indicated by magenta arrows. The phenylalanine conserved in the bacterial structures possibly aiding in positioning in aromatic substituents of the sugar esters is indicated with a grey arrow. The disulfide bridges formed in the fungal structures are indicated above the alignment by numbering in green text.


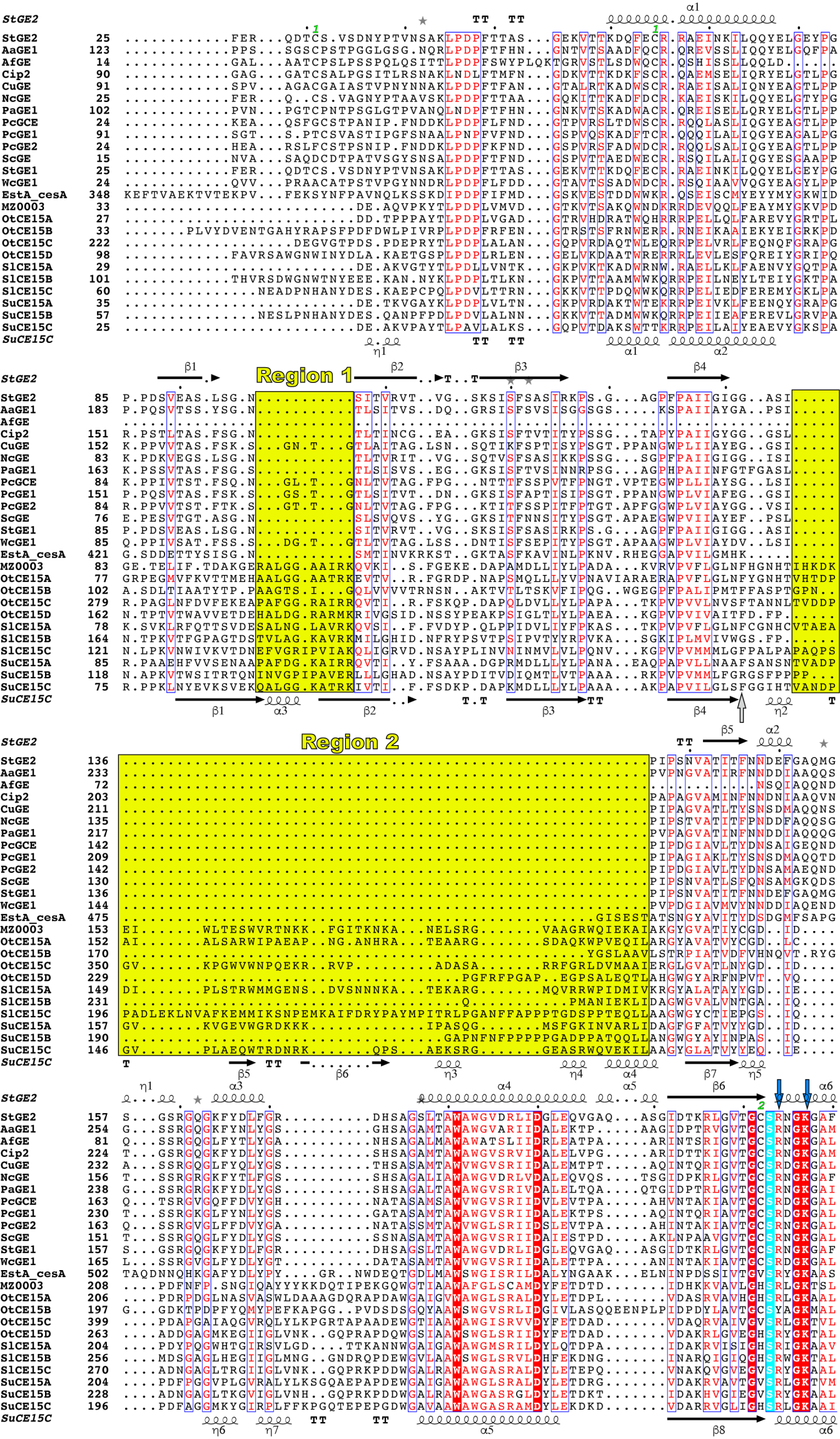

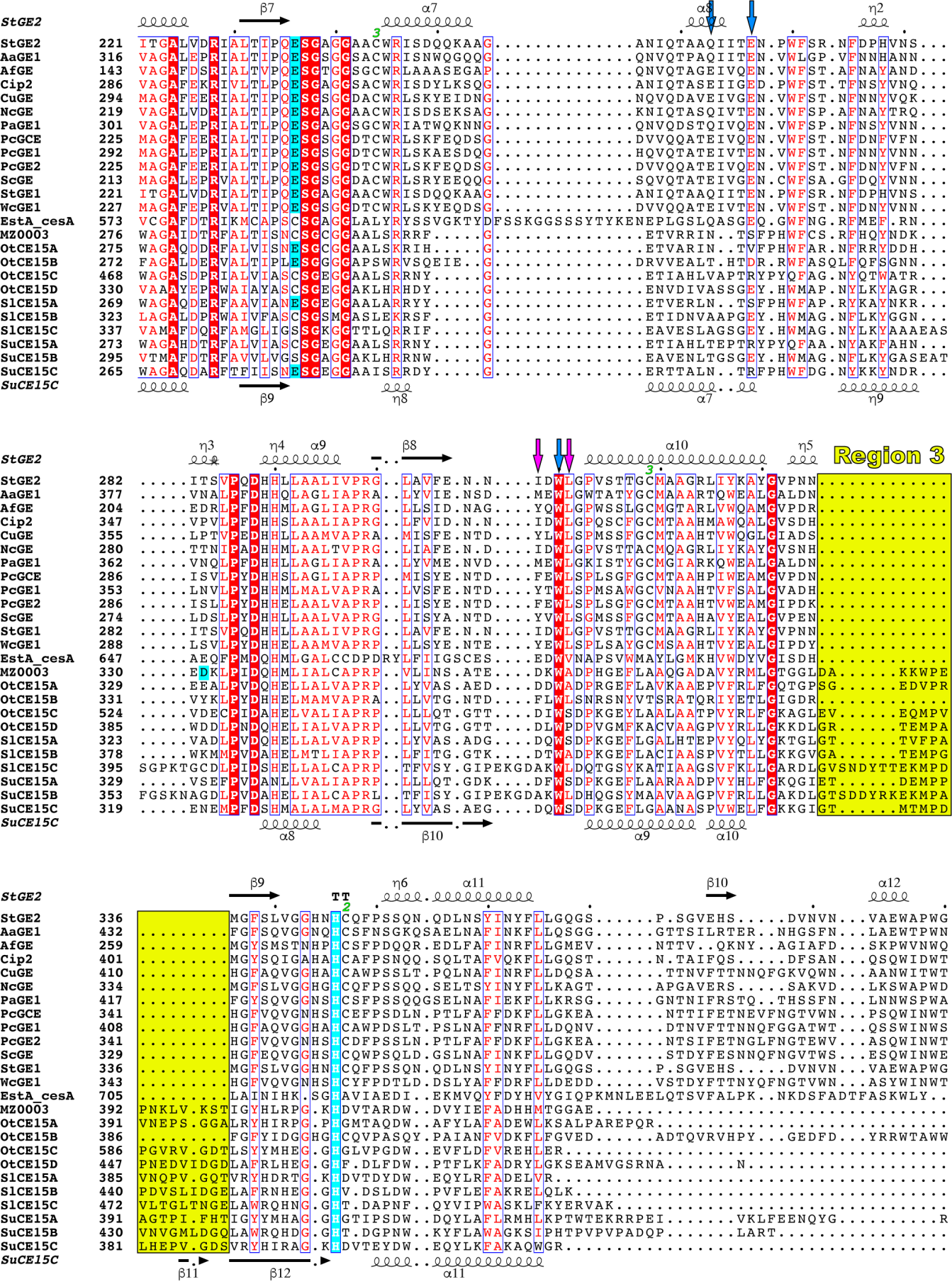


**Figure S5.** Multiple sequence alignment of characterized glucuronoyl esterases. Similar residues are written in red text while conserved residues are written in white text over a red background. The insertion regions found in the bacterial structures relative to the fungal counterparts are highlighted in yellow. The residues of the conserved catalytic triad are colored cyan. Note that glutamate of the catalytic triad is not conserved in all bacterial esterases and the position of the equivalent acidic residue in MZ0003 is also colored cyan. Arrows indicating significant residues are colored as in Figure S4.


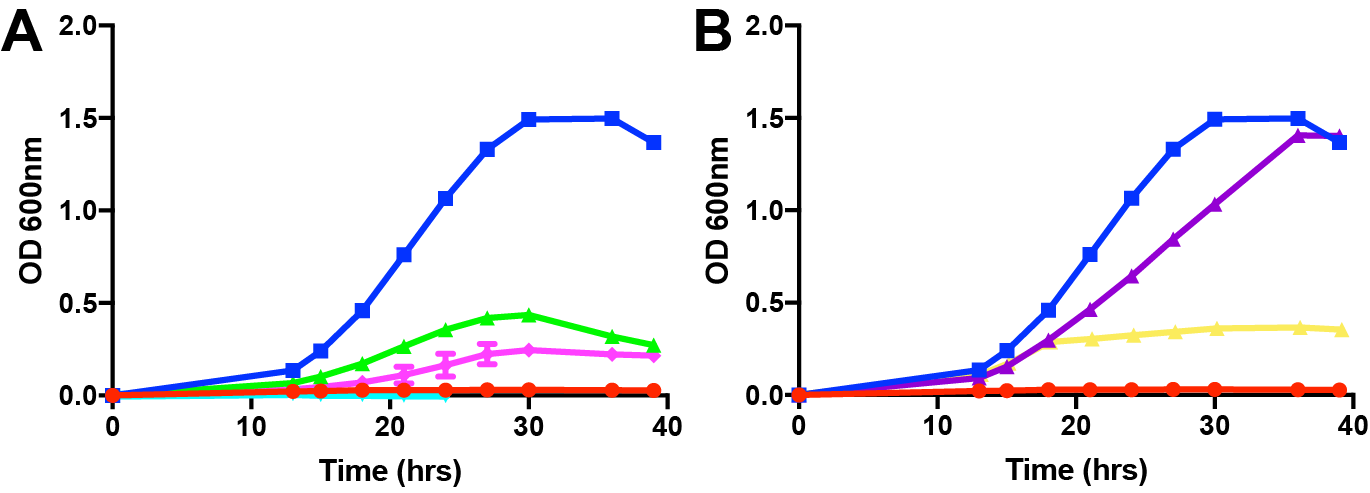


**Figure S6.** Growth curves of *S. linguale* when grown with different additives or on different carbon sources. *S. linguale* did not grow on standard minimal media and an optimized media for bacterial growth was determined experimentally (see methods for formulation). (A) Growth curves of *S. linguale* in the optimized media without a carbon source (red), with 0.3% (w/v) glucose (blue) and in the media containing glucose but in the absence of either trace metals and vitamins (green), sodium phosphate pH 7.5 (magenta), or magnesium sulphate (cyan). (B) Growth of *S. linguale* in optimized media with 0.3% of either glucose (blue), xylose (purple), or xylan from corn cob (yellow).
